# Archimedes’ principle for characterisation of recombinant whole cell biocatalysts

**DOI:** 10.1038/s41598-018-20877-1

**Published:** 2018-02-14

**Authors:** Steven Schmitt, Marcel Walser, Michael Rehmann, Sabine Oesterle, Sven Panke, Martin Held

**Affiliations:** Department of Biosystems Science and Engineering, ETH Zürich, Mattenstrasse 26, 4058 Basel, Switzerland

## Abstract

The ability of whole cells to catalyse multistep reactions, often yielding synthetically demanding compounds later used by industrial biotech or pharma, makes them an indispensable tool of synthetic chemistry. The complex reaction network employed by cellular catalysts and the still only moderate predictive power of modelling approaches leaves this tool challenging to engineer. Frequently, large libraries of semi-rationally generated variants are sampled in high-throughput mode in order to then identify improved catalysts. We present a method for space- and time-efficient processing of very large libraries (10^7^) of recombinant cellular catalysts, in which the phenotypic characterisation and the isolation of positive variants for the entire library is done within one minute in a single, highly parallelized operation. Specifically, product formation in nanolitre-sized cultivation vessels is sensed and translated into the formation of catalase as a reporter protein. Exposure to hydrogen peroxide leads to oxygen gas formation and thus to a density shift of the cultivation vessel. Exploiting Archimedes’ principle, this density shift and the resulting upward buoyancy force can be used for batch-wise library sampling. We demonstrate the potential of the method for both, screening and selection protocols, and envision a wide applicability of the system for biosensor-based assays.

## Introduction

Screening and selection procedures are key strategies in the discovery or optimization of whole cell biocatalysts in high-throughput campaigns. In both cases, changes in the concentration or the activity of the product need to be read out. For screening assays, improved strains are typically identified by a colour change, or an increased fluorescence or luminescence signal, while in the case of selection assays, positive variants show an elevation of the growth rate over the background of negative variants^1^. Frequently, analysis and sorting of the samples occur in a consecutive fashion in which samples are analysed one after the other, and then a mechanical operation is applied to separate a potentially positive variant from the rest. As a result, the throughput remains limited. Methods enabling high throughput at reasonable costs are therefore in high demand^2–5^.

We exploited Archimedes’ principle, which relates the buoyancy force of an immersed object to its density, to identify positive variants in biocatalyst screening and selection assays. By changing the buoyancy force of a cultivation vessel with a monoclonal microcolony in response to a desired change, such vessels can be made to ascend from a background population and float, allowing for strictly parallel analysis and sorting of strain variants at very high rates. As cultivation vessels, we used monodisperse hydrogel-based nanolitre reactors (nLRs), with a precisely adjustable diameter between 200 to 500 µm, as microcompartments for controlled expansion of single cells into monoclonal microcolonies (approx. 10 to 100 µm colony diameter), analysis, and recovery of positive variants^6–9^.

## Results and Discussion

We reasoned that in order to readily separate an nLR containing a microcolony of a desired strain variant from a background of nLRs, we should aim for a difference in buoyancy of at least 20%, i.e. a reduction of the density of approx. 1.1 g mL^−1^ for an nLR containing mostly water and cells^10^ to less than 0.9 g mL^−1^. Different possibilities were considered (see Supplementary Notes), but we found enzymatically catalysed gas formation from dissolved precursors most promising. Indeed, heterologous production of catalase (EC 1.11.1.6, from *Listeria seeligeri*)^11^ in *Escherichia coli* MDS42 from plasmid pCat led to liberation of molecular oxygen at rates of approx. 9 pL min^−1^ cell^−1^ (see Supplementary Fig. 1) from externally added hydrogen peroxide. Please note that the amount of gas released by a single cell in one minute exceeds the volume of the cell approximately 5,000-fold.

**Figure 1:**
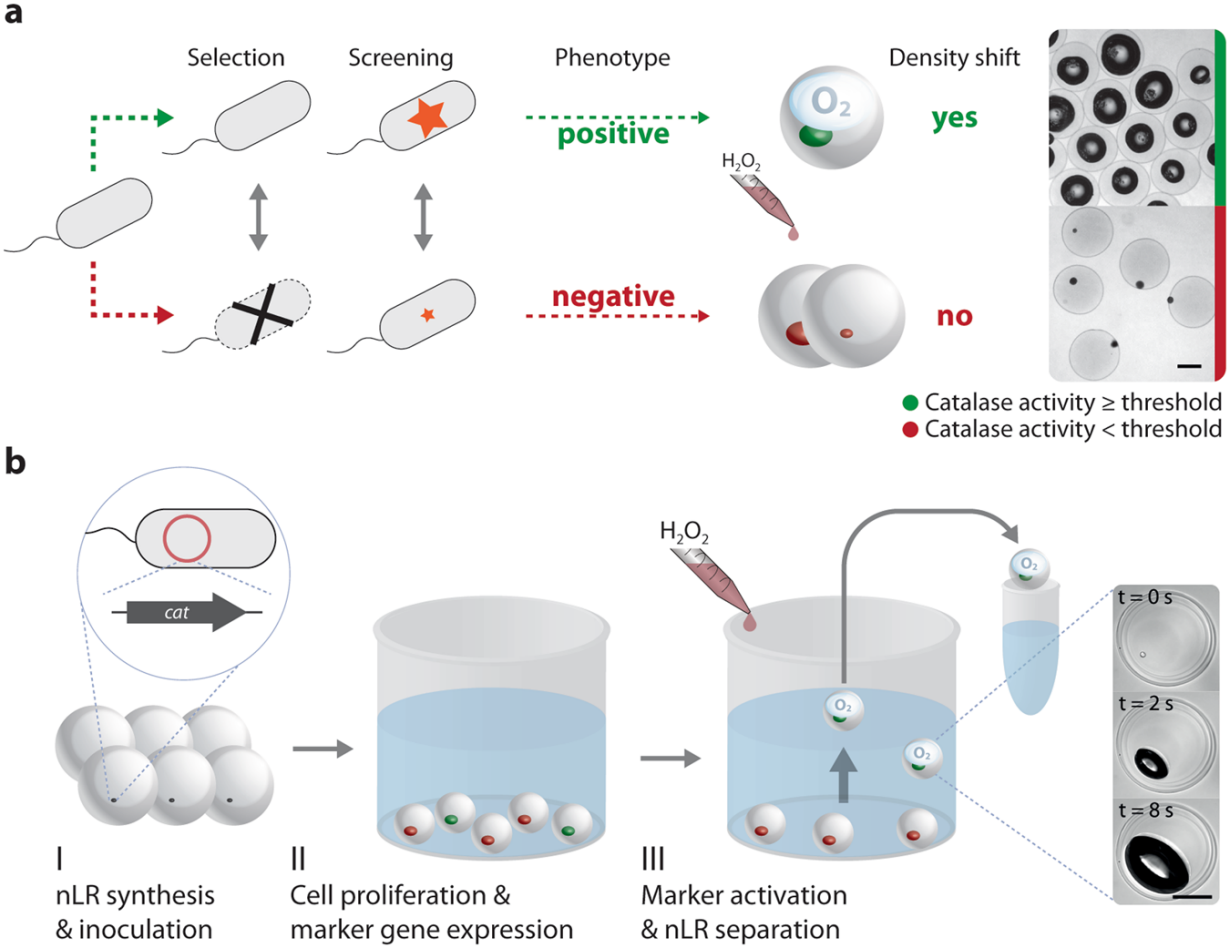
High-throughput phenotyping of whole cell biocatalysts employing buoyancy modulation markers. (**a**) Assays based on selection or on screening can be adapted to buoyancy separation. In both cases, the amount of catalase marker in an nLR needs to become larger than a critical threshold in order to make an nLR (and the strain variant residing within) ascend. This can be achieved by varying the biomass level (selection scenario) or the expression level (screening scenario). Scale bar: 200 µm. (**b**) Separation workflow. (I) Single library cells are encapsulated into nLRs. (II) The nLRs are incubated in growth medium until microcolonies are formed. (III) H_2_O_2_ is added and microcolony-containing nLRs with a catalase amount beyond the threshold ascend within a few seconds due to the formation of an O_2_-filled gas chamber within their interior (see inset and Supplementary Video 1). Scale bar: 150 µm.

When nLRs containing microcolonies of *E*. *coli* MDS42 [pCat] were incubated in diluted H_2_O_2_, the ensuing rapid gas production led to the formation of O_2_-filled chambers inside the nLRs. These chambers were formed within seconds, showed considerable stability (≫10 min) and primarily expanded towards the inner core of the nLRs, possibly following the density gradient of the hydrogel^12^ (see Fig. 1a, Supplementary Video 1). The gas occupied >30% of the nLR volume and thus induced a sufficiently large density decrease to allow them ascending to the top of an aqueous liquid (see Fig. 1b, Supplementary Fig. 2 and Supplementary Video 2). Despite the considerable cytotoxicity of H_2_O_2_, we could recover living cells from more than 90% of the H_2_O_2_-treated (2% H_2_O_2_, ≤5 min contact time) colonies. When reducing the contact time and concentration of H_2_O_2_ to the minimum necessary for buoyancy separation, all colonies could be regrown after the separation process (see Supplementary Fig. 3).

The magnitude of the density shift is expected to primarily depend on the amount of catalase per nLR, making this a potentially quantifiable marker in screening and selection. To this end, both the specific growth rate of a strain (and therefore the size of a microcolony at the time of assaying) and the catalase expression level can be exploited to make nLRs ascend (see Fig. 1 and Supplementary Fig. 4).

**Figure 2:**
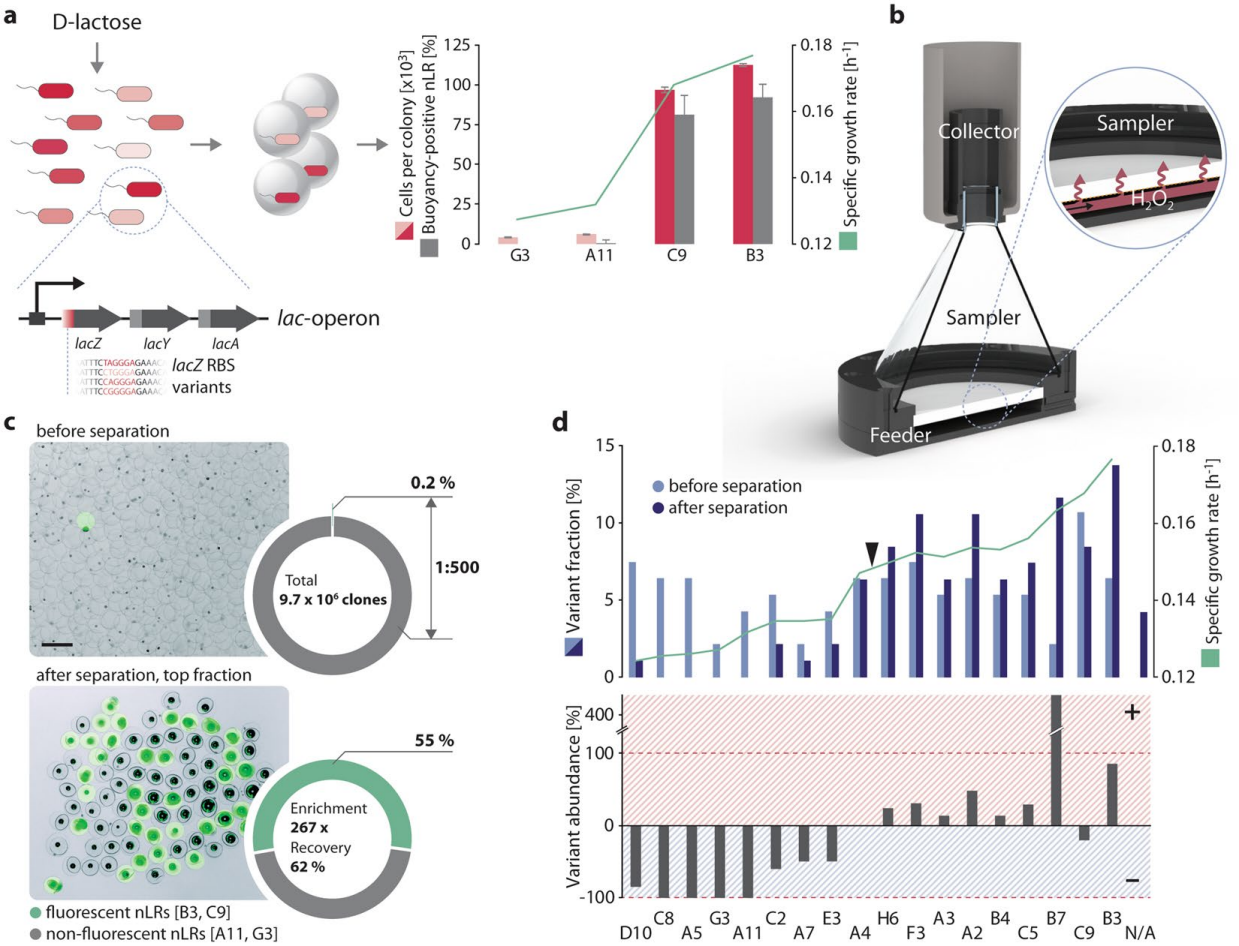
Buoyancy separation of whole cell biocatalysts according to their growth rate. (**a**) The specific growth rate of *E*. *coli* EcNR1 variants on d-lactose was determined. Cells from two slow- (G3, A11) and two fast-growing (C9, B3) variants were expanded to microcolonies in nLRs. In the presence of H_2_O_2_, the majority of microcolonies formed from the fast-growing cells produced ascending nLRs (C9: 139 of 171; B3: 156 of 170) whereas the slow-growing cells did not (G3: 0 of 157; A11: 1 of 150). One-way ANOVA indicated a statistically significant difference among the different strains for both cells per colony (F(3,745) = 276.80, p < 0.00001, α = 0.01) and separation (F(3,32) = 373.25, p < 0.00001, α = 0.01). Data shown as mean ± SD, n = 9 with 14 to 21 nLRs per experiment. (**b**) Sectional drawing of the device used for separation (see also Supplementary Fig. 8). (**c**) The nLRs containing slow-growing strains were incubated in approx. 500-fold excess together with fast-growing strains until microcolonies had formed. Then, 1.73 × 10^7^ nLRs (with a total of 9.72 × 10^6^ microcolonies) were buoyancy-separated using the device shown in b. The nLRs containing the fast-growing variants B3 and C9 also carried a fluorescence label which allowed identifying the genotype by epifluorescence microscopy before (upper picture) and after (lower picture) separation (GFP filter set, see Supplementary Methods). A sample of 20,000 nLRs of the initially prepared total population and the entire buoyancy-positive top fraction (22,585 nLRs) were analysed by large particle sorting to determine the enrichment factor. Scale bar: 400 µm. (**d**) A total of approx. 16,000 cells representing 18 strain variants with different growth rates were distributed over approx. 50,000 nLRs and subjected to buoyancy separation after incubation. Specific genotypes in a subset (48 clones of each subset) of the total population before and after separation were determined (upper graph) and used for the calculation of the variant abundance (variants after separation x variants before separation^−1^ − 1, lower graph). The triangle indicates the specific growth rate of unmodified *E*. *coli* EcNR1. N/A: Samples with more than one genotype per nLR (e.g. nLRs that had received two or more library cells during inoculation).

We first showed that buoyancy-triggered separations can be used for selection assays. As a mock library, we generated *E*. *coli* EcNR1 variants^13,14^, each metabolizing d-lactose at a different rate and thus displaying different maximum specific growth rates (µ = 0.12 to 0.18 h^−1^ on chemically defined medium with d-lactose as the sole carbon source, see Fig. 2a and Supplementary Fig. 5) ^15,16^. We induced expression of the catalase marker from pCat to a level corresponding to an O_2_ release of approx. 300 fL O_2_ min^−1^ cell^−1^. Next, two slow- and two fast-growing isolates (G3, µ = 0.127 h^−1^; A11, µ = 0.132 h^−1^; C9, µ = 0.168 h^−1^; and B3, µ = 0.177 h^−1^, see Supplementary Fig. 6) were proliferated in nLRs for the same amount of time. The nLRs containing differently sized microcolonies were then buoyancy-separated on microscope slides in a water droplet (see Fig. 2a). Microscopic observations indicated a good correlation between the size of the microcolony (i.e., the number of cells in an nLR) and the number of ascending nLRs.

Next, a simple, customized device for buoyancy separation of larger batches of nLRs (see Fig. 2b and Supplementary Fig. 8) was implemented. A total of 1.73 × 10^7^ nLRs containing 9.72 × 10^6^ microcolonies (4.85 × 10^6^ microcolonies of each of the slow-growing variants, G3 and A11, and 10,000 of each of the fast-growing variants, C9 and B3) were treated with H_2_O_2_. Positive and negative nLRs separated within one minute. The nLRs containing fast-growing strains had also been fluorescently labelled, thereby allowing counting by large-particle flow cytometry^6^. The results indicated a 267-fold enrichment of the fast-growing over the slow-growing variants (from 0.2% to 55% of occupied nLRs) and a recovery of 62% (12,426 nLRs with fast-growing strains were recovered out of an initial total of 20,000; see Fig. 2c). Indeed, sequencing of 48 clones recovered from the isolated fluorescent nLRs indicated that only fast-growing variants had been isolated.

Next, we pooled 18 strains displaying different growth rates, used them for nLR inoculation (approx. 50,000 nLRs inoculated with 16,000 cells), incubated the nLRs to generate microcolonies, and subjected them to buoyancy separation. Microscopic inspection before and after separation indicated the absence of large microcolonies in the buoyancy negative bottom fraction (see Supplementary Fig. 7) while genotyping of microcolonies obtained from the buoyancy-positive top fraction showed efficient enrichment of nLRs harbouring fast-growing variants (see Fig. 2d). In summary, these results suggest that selection assays can be readily adapted to protocols relying on buoyancy separation as readout.

**Figure 3:**
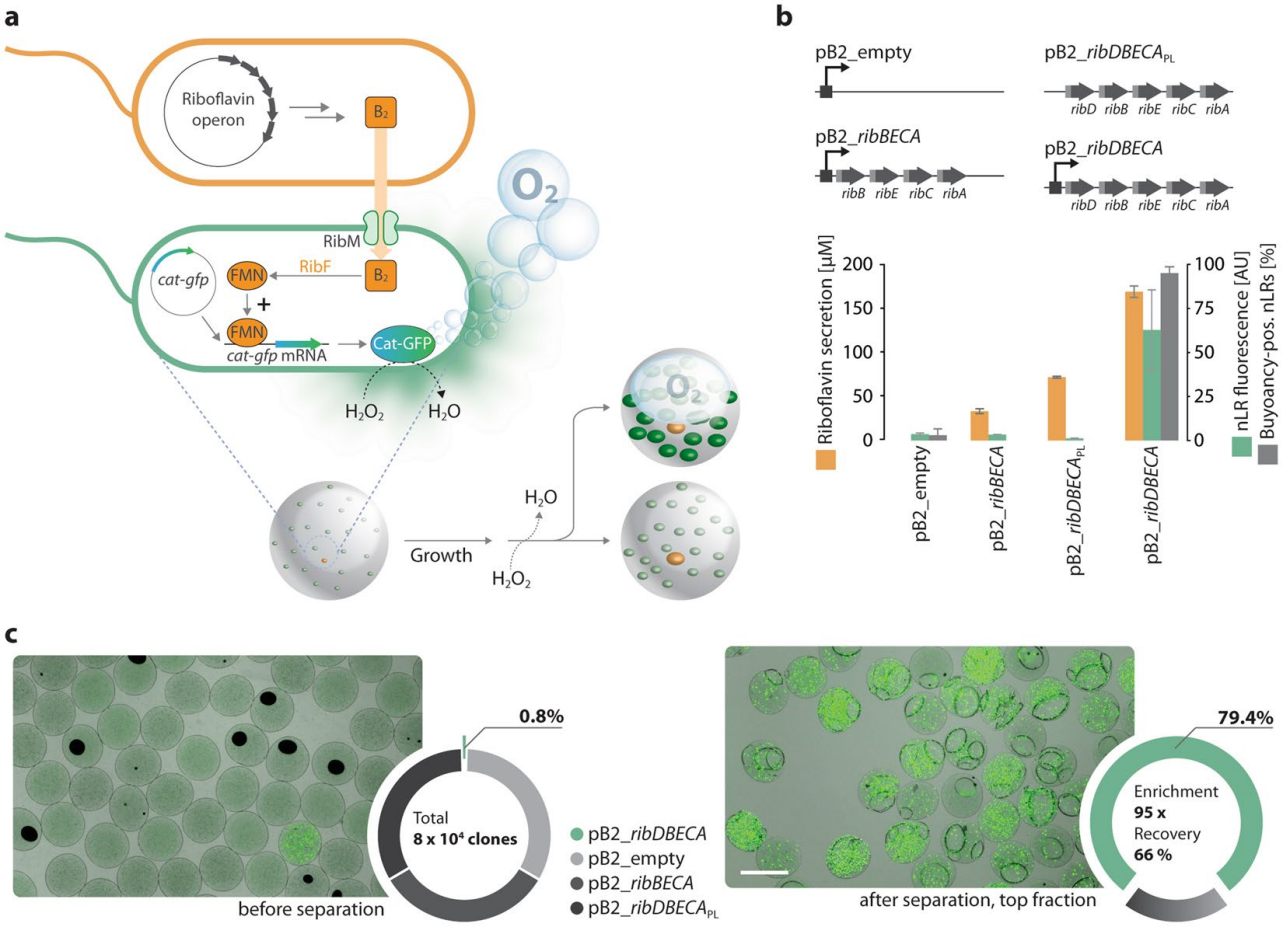
Enrichment of high B_2_ producing strains by buoyancy separation. (**a**) Principle of the screen: The nLRs are inoculated with B_2_ producing (orange) and B_2_ sensing (green) *E*. *coli* cells. The sensing cells take up B_2_ via RibM and convert it to FMN. FMN then translationally upregulates synthesis of a Cat-GFP fusion protein, thereby providing the basis for two-fold identification of potent B_2_ secreting isolates. (**b**) Upper panel: *E*. *coli* BW23474 whole cell biocatalysts were equipped with one of four different plasmids leading to different degrees of B_2_ overproduction in shake flasks (lower panel, orange columns). Next, the biocatalysts were encapsulated in nLRs (on average 0.2 cells per nLR) together with biosensors (on average 1,000 cells per nLR). The nLRs were incubated, subjected to separation on a microscope slide in a water droplet, and the buoyancy-positive nLRs were counted under the microscope and analysed for GFP fluorescence. Only the high B_2_ producing strain led to a large fraction of ascending nLRs (pB2_*ribDBECA*: 114 of 120), whereas the others did not (pB2_*ribDBECA*_PL_: 0 of 60; pB2_*ribBECA*: 0 of 57; pB2_empty: 3 of 101). One-way ANOVA indicated a statistically significant difference among the different strains for B_2_ secretion (F(2,15) = 1312.57, p < 0.00001, α = 0.01) and separation (F(3,14) = 1463.94, p < 0.00001, α = 0.01). Data shown as mean ± SD, n = 4 to 5 with 11 to 25 nLRs per experiment. (**c**) Approx. 363,000 nLRs were inoculated with approx. 79,000 *E*. *coli* cells obtained from four different B_2_ producing whole cell biocatalysts (see b). The most potent B_2_ producing whole cell biocatalyst carrying plasmid pB2_*ribDBECA* was underrepresented (660 cells, 0.8%). An overlay of bright field and epifluorescence microscopic images of the nLR population before (left image, bulk) and after (right picture, top fraction) buoyancy separation indicates efficient enrichment of nLRs with Cat-GFP overexpressing biosensors. These observations were verified by counting the fluorescently labelled high B_2_-producer containing nLRs within the total population by large-particle flow cytometry (549 nLRs). Scale bar: 500 µm.

In the case of screening assays, the growth rate of cells is usually similar but differences in a desired property (e.g. product formation) lead to differences in the expression level of a marker protein. We used a riboswitch-based control element^8^ for translational control of catalase production in response to the industrial product vitamin B_2_ (riboflavin) secreted by whole cell biocatalysts^17^. First, a collection of biocatalysts with different B_2_ production capacities was generated. For this, *E*. *coli* BW23474 was transformed with plasmids containing either the synthetic B_2_ biosynthesis operon *ribDBECA* (expressing all genes required for the synthesis starting from GTP and ribulose-5-phosphate and driven by a recombinant promoter), the operon *ribDBECA*_*PL*_ (no recombinant promoter), or the operon *ribBECA* (missing *ribD* and therefore potentially undersupplying the important intermediate 5-amino-6-(5′-phospho-ribitylamino) uracil; see Supplementary Fig. 9). The respective whole cell biocatalysts secreted B_2_ to levels of up to 170 µM (pB2_*ribDBECA*), 72 µM (pB2_*ribDBECA*_*PL*_), and 33 µM (pB2_*ribBECA*) (see Fig. 3b).

Next, a biosensor for the translation of different B_2_ levels into different catalase amounts was constructed. The biosensor cells use the B_2_ transporter RibM for facilitated B_2_ uptake^18^ and convert it to flavin mononucleotide (FMN) by the endogenous enzyme RibF. Elevated B_2_ levels thus lead to elevated FMN levels, which lead in turn to the increased expression of Cat-GFP, a fusion of the catalase enzyme to the N-terminus of the superfolder green fluorescent protein (sfGFP), via an FMN-sensitive hammerhead ribozyme^8^ (Fig. 3a). While the catalase domain of the fusion relates nLR ascension to vitamin production levels, the sfGFP enables ascension-independent tracking of the catalase expression level.

To test the system, nLRs were co-inoculated with the different B_2_ producer and biosensor cells such that each nLR contained on average 0.3 producer and 1,000 biosensor cells. The nLRs were soaked with growth medium and incubated in a hydrophobic phase in order to avoid inter-nLR exchange of nutrients or B_2_. Once the B_2_ producing cells had proliferated to microcolonies, the expression level of the Cat-GFP fusion protein in the biosensor cells was measured by large-particle flow cytometry. The results indicate high Cat-GFP levels for the strain that carried the full *ribDBECA* operon controlled by a recombinant promoter and therefore secreted high levels of B_2_ (Fig. 3b). Next, we analysed the ascension of the colonized NLRs under the microscope. Only nLRs carrying the high B_2_ producing strain ascended, indicating a good correlation between fluorescence levels and catalase activity (see Fig. 3b and Supplementary Fig. 10). For a large-scale test, 660 nLRs carrying a microcolony of the high B_2_ producing strain harbouring plasmid pB2_*ribDBECA* and a fluorescent label (in order to facilitate subsequent identification) were mixed with a background of 363,000 nLRs containing 79,000 *E*. *coli* microcolonies of the poorly performing strains carrying the plasmids pB2_*empty*, pB2_*ribBECA*, and pB2_*ribDBECA*_*PL*_. The resulting mixture was subjected to buoyancy separation and the buoyancy-positive top fraction consisting of 549 nLRs was isolated. Microscopic analysis revealed that 472 were fluorescently labelled and therefore carrying the potent B_2_ producer (pB2_*ribDBECA*). The results indicate a 95-fold enrichment (from 0.8% to 79%) and a recovery of 66% (436 of 660; see Fig. 3c). A fraction (96 nLRs) of the isolated nLRs was spotted on solidified medium that would allow growth of the producer but not of the biosensor. 86% of nLRs containing a high B_2_-producing clone could be re-grown. This translated into a recovery of 57% over all process steps and clearly illustrates the suitability of the method for screening assays.

In summary, application of Archimedes’ principle allows for highly parallelized analysis and separation of genetically distinct whole cell biocatalysts in a single step. We demonstrated the application of buoyancy separation using catalase as a marker in two conceptually different settings frequently applied for optimization of whole cell biocatalysts, (I) selection and (II) screening. We showed that either differences in metabolic activity or expression level of a marker protein can be used to produce differential buoyancy forces, resulting in a clear-cut separation of diversely performing whole cell biocatalysts. This suggests good compatibility with the existing toolbox of biological sensing elements including RNA^19,20^ or regulator protein-based circuits^21,22^, and specifically engineered sensor strains^23,24^. The straight forward replacement of commonly used fluorescent proteins (e.g. GFP) by the catalase protein renders such tools suitable for buoyancy separation. Using a simple lab-demonstrator, we analysed and isolated 10^7^ samples within one minute, hence exceeding the throughput at which standard flow-cytometric sorters operate by at least one order of magnitude. We further argue that the capacity of the method can be adapted by changing the diameter of the customized separator such that scaling up to 10^9^ analysed microcolonies per batch would require only a small adaptation (see Supplementary Notes). Further increase of throughput can be achieved by reduction of the nLR size and increasing the frequency of production (see Supplementary Notes). The nLR generator employed in this study produces between 700 and 4,000 nLRs per second and generates compartments between 200 and 500 µm, but microfluidic- or bulk-emulsification processes enable even higher throughput (>10,000 nLRs per second) at smaller diameters (<100 µm)^25–29^. Together, both changes suggest that an increase of the analysis and isolation rates to more than 10^10^ nLRs per batch and minute is possible.

## Methods

### Construction of the catalase marker

The catalase marker from *L*. *seeligeri* was isolated from plasmid pAHA1 (see Supplementary Table 3 for a comprehensive list of plasmids), kindly provided by Albert Haas (University of Bonn, Germany) using the restriction enzymes NdeI and NsiI. To allow for stringent transcriptional control, the catalase gene was placed under control of the P_tet_ promoter cassette^30^. The promoter, together with the gene for the corresponding regulator TetR, was excised from plasmid pKTS using BamHI and NdeI. Note that the cassette additionally contains a T7 promoter that was not used in our experiments. The catalase gene and promoter were joined by ligation and cloned into plasmid pAct3, linearized with NsiI and BamHI to give plasmid pCat. Correct fusion of promoter and gene was verified by Sanger sequencing using primer ID 1 (see Supplementary Table 1 for a comprehensive list of oligonucleotides). *In vivo* characterization of the catalase marker was performed by transformation of *E*. *coli* MDS42 obtained from Scarab Genomics (Madison, WI, see Supplementary Table 2 for a comprehensive list of strains) with plasmid pCat.

### Ribosome binding site (RBS) modification

The RBS of the *lacZ* gene in *E*. *coli* EcNR1 (kindly provided by George Church, Harvard University, MA) was modified using the CRMAGE^31^ strategy. Briefly, an oligonucleotide pool (oligonucleotide ID 8) introducing changes within the wild-type RBS of *lacZ* was used for MAGE^13^. The pool contained 18 different oligonucleotides, all targeting the RBS of the *lacZ* gene, but each of them encoding a different RBS-sequence (see Supplementary Fig. 5). During MAGE, counterselection against cells retaining the wild-type RBS was performed using the CRIPSR/Cas9^14^ system. Plasmids pCRISPR and pCas9 were kindly provided by Luciano Marraffini (The Rockefeller University, NY). The gRNA for targeting Cas9 to the wild-type RBS sequence was designed and checked for off-target effects with the Cas9 Online Designer (COD)^32^ and the resulting oligonucleotides encoding the gRNA sequence (oligonucleotides ID 6 and ID 7) were annealed and ligated into BsaI-linearized pCRISPR plasmid to obtain plasmid pCRISPR_*lacZ*. Chromosomal modifications were performed in strain *E*. *coli* EcNR1, previously transformed with plasmid pCas9. Briefly, a 3 mL culture was grown in LB-Luria broth (as LB-Miller, but salt reduced to 0.5 g L^−1^ NaCl), supplemented with 20 µg mL^−1^ chloramphenicol, and incubated at 32 °C until an OD_600_ of approx. 0.6 was reached. Cells were then heat-shocked for 15 min at 42 °C to induce expression of the λ-Red genes. Next, the induced cells were made electrocompetent by washing three times with ice-cold ddH_2_O and resuspended in a volume of 50 µL. The *lacZ* RBS oligonucleotide pool was then added to a final concentration of 2 µM and the cells were electroporated in 1 mm gap cuvettes (Cell Projects, Harrietsham, United Kingdom) at 1.8 kV and 4 to 6 ms pulse-length. Cells were recovered by addition of 3 mL LB-Luria broth supplemented with 20 µg mL^−1^ chloramphenicol, again induced at 42 °C and made competent for one further electroporation with the oligonucleotide pool where 100 ng of the pCRISPR_*lacZ* plasmid was also added. Cells were then recovered overnight in LB-Luria broth, supplemented with 50 µg mL^−1^ kanamycin (added 1 h after electroporation) and plated the next day on LB agar plates with with 50 µg mL^−1^ kanamycin. To identify clones with the correct RBS modification, the genomic region containing the *lacZ* RBS was amplified by colony PCR using *Taq* DNA polymerase and primers ID 9 and ID 10 and the PCR product was Sanger sequenced using DNA oligonucleotide ID 9. For selected strains, the plasmid pCas9 was removed from the cells by repetitive cultivation without antibiotic selection (two times, followed by replica plating to verify loss of the chloramphenicol resistance). The strains were transformed with the plasmid pCat to enable catalase-based separation, resulting in 18 different strains *E*. *coli* EcNR1_XX [pCRISPR_*lacZ* + pCat] (where XX represents the ID of the RBS variant).

### B_2_ biosensor engineering

*E*. *coli* BW25113 served as a chassis for the construction of the B_2_ auxotrophic biosensor. The strain was engineered using plasmid-based homologous recombination^33^. Briefly, a part of the chromosomal gene for the riboflavin synthase RibC was replaced by the gene for the riboflavin transporter RibM (PnuX) from *Corynebacterium glutamicum*^18^. Plasmid pKO3_*ribM* contained a 2.5 kbp fragment amplified from the genome of *E*. *coli* JM101 encoding for the *ribC* gene. While deleting 247 bp of the central part of *ribC*, the *ribM* gene was integrated into that sequence together with a RBS to allow for *ribM* translation. The plasmid was used to transform *E*. *coli* BW25113 and homologous recombination was performed according to the protocol provided by Link *et al*.^33^ but additionally 10 µM B_2_ was added to the medium. The resulting strain *E*. *coli* BW25113 Δ*ribC*::*ribM* is a B_2_ auxotroph but can grow with externally supplied B_2_ taken up via RibM. Correct insertion of the *ribM* gene was verified by colony PCR using primers ID 11 and ID 12 and replica plating on LB-Miller agar plates with and without B_2_.

The previously constructed expression system pRSG_2A1 was used for regulation of catalase expression. Briefly, plasmid pRSG_2A1 contains a sfGFP gene under transcriptional control of the P_*tac*_ promoter. The translation of the resulting mRNA is controlled by an aptazyme consisting of FMN aptamer 21A^8^, an optimized connecting linker and a hammerhead ribozyme. To adapt it for the expression of the catalase, plasmid pRSG_2A1 was linearized by cutting between the gene for the aptazyme and *sfgfp* with the restriction enzyme NsiI. The catalase gene was PCR-amplified from plasmid pCat using primers ID 13 and ID 14, digested with NsiI and ligated into the linearized pRSG_2A1, thus generating a plasmid containing a translational fusion of the catalase to the N-terminus of sfGFP (Cat-GFP). Correct clones were identified by testing for their fluorescence properties and catalase activities and confirmed by Sanger sequencing (primers ID 15 and ID 16). Next, the *cat*-*gfp* gene together with its regulatory elements was isolated by restriction digestion with NdeI and HindIII and cloned into plasmid pSEVA281, linearized with the same enzymes, to generate plasmid pSense_*cat*-*gfp*. The plasmid was then used to transform the strain *E*. *coli* BW25113 Δ*ribC*::*ribM* yielding the final B_2_ biosensor.

Prior to its application as B_2_ biosensor, the strain *E*. *coli* BW25113 Δ*ribC*::*ribM* [pSense_*cat*-*gfp*] was grown in LB-ΔRib broth (inoculated 1:100 from a pre-culture in stationary phase; for comprehensive description of media and buffer compositions, please see Supplementary Information). Culturing was continued until the cells became B_2_ limited and did not grow anymore even in the presence of excess nutrients (usually two sub-cultivations). Then, the B_2_ limited cells were supplemented with glycerol to a final volume fraction of 20% and stored as frozen stocks at −80 °C.

### Synthesis of nLRs

Nanolitre reactors (nLRs) were synthesized as described previously^8^. Single droplets were generated by laminar-jet breakup with a Var D encapsulator (Nisco Engineering AG, Zürich, Switzerland) from sodium alginate suspensions (20 g L^−1^ in ddH_2_O) inoculated with a precisely adjusted concentration of bacterial cells (see results). Inoculation was either done from a liquid pre-culture, where the number of colony-forming units (CFUs) per volume was estimated by measuring the optical density at a wavelength of 600 nm (1 OD_600_ unit = approx. 5 × 10^8^ CFUs mL^−1^) using a BioPhotometer (Eppendorf, Hamburg, Germany), or from frozen-stocks of the strains, where the number of CFUs was measured by plating and colony counting on agar plates beforehand. The desired number of cells were added to one part of sterile ddH_2_O in a 50 mL centrifugation tube and filled up with four parts of sodium alginate (25 g L^−1^ in ddH_2_O). When fluorescently labelled nLRs were required, fluorescein-alginate (from a 5 g L^−1^ stock-solution in ddH_2_O, see Supplementary Methods) or a ROX-silica-particle suspension (from a 3 g L^−1^ stock-solution in ddH_2_O, see Supplementary Methods) was added to a final volume fraction of 0.5% together with the cells to the water. After mixing by inverting the tube several times, the suspension was transferred into a 24 mL or 60 mL syringe and connected to the syringe pump of the encapsulation device. Depending on the desired size of the nLRs, one of the following encapsulator operation mode was selected: Production of nLRs with 460 µm diameter (approx. 50 nL volume): 0.7 kHz with 3.3 mL min^−1^ alginate flow-rate and a nozzle diameter of 150 µm. Production of nLRs with 230 µm diameter (approx. 6 nL volume): 3.75 kHz with 2.0 mL min^−1^ alginate flow-rate, coaxial-nozzle with an outer diameter of 350 µm and operated at an airstream with a pressure of 30 mbar. Reactors were gelled in nLR hardening buffer for 20 min and briefly rinsed with nLR wash buffer before transferring them into growth medium.

### Cultivation of cells in nLRs

#### Preparation of nLRs for the characterization of catalase-based separation

For initial characterization of the buoyancy properties, *E*. *coli* strain MDS42 [pCat] was grown from frozen stocks in 5 mL LB-Miller broth (supplemented with 20 µg mL^−1^ chloramphenicol) in a pre-culture until mid-exponential phase (OD_600_ = 0.6 to 1), encapsulated into nLRs with a diameter of approx. 460 µm and incubated (100 g L^−1^ wet nLRs) in Erlenmeyer shake-flasks in OB broth (4 g L^−1^ yeast extract, 1 g L^−1^ tryptone, 1 g L^−1^ glycerol, 2 mM CaCl_2_, 10 mM Tris-HCl, pH 7). Incubation was performed at 27 °C (specific growth rate of approx. 0.44 h^−1^) or 30 °C (approx. 0.54 h^−1^) on an orbital shaker (160 rpm, 25 mm amplitude, Kuhner, Birsfelden, Switzerland). The induction of catalase gene expression was initiated after 18 h of incubation with 0 to 200 ng mL^−1^ anhydrotetracycline. After 22 h incubation was stopped, nLRs were washed several times with nLR wash buffer and stored at 4 °C in the same buffer until separation.

#### Preparation of nLRs with lacZ RBS variants for buoyancy separation

The *E*. *coli* EcNR1_XX [pCRISPR_*lacZ* + pCat] strains containing the different *lacZ* RBS variants were grown from frozen stocks in two consecutively inoculated pre-cultures (both supplemented with 20 µg mL^−1^ chloramphenicol and 50 µg mL^−1^ kanamycin): (I) in LB-Miller broth and (II) in CDM (see Supplementary Methods for composition) supplemented with 4 g L^−1^ D-glucose. Culturing was performed in 800 µL of medium in Nunc DeepWell 96-well plates. After encapsulation, nLRs were incubated (50 g L^−1^ wet nLRs) in Erlenmeyer shake-flasks containing CDM supplemented with 4 g L^−1^ D-lactose, 20 µg mL^−1^ chloramphenicol and 50 µg mL^−1^ kanamycin at 30 °C on an orbital shaker (160 rpm, 25 mm amplitude). In cases where the precise number of occupied nLRs was important, nLRs were incubated for 5 h under conditions described above, isolated into nLR wash buffer and the embedded microcolonies (consisting of approx. 1 to 10 cells) were stained with the DNA intercalating green fluorescent dye SYTO 9 (Thermo Fisher Scientific, Waltham, MA). Next, monoclonally occupied nLRs were isolated by large-particle flow cytometry (based on the fluorescence of the contained microcolonies), the sorted nLRs were then transferred to the same medium again and incubation was continued. If co-incubation of different strains was desired, batches of counted nLRs were mixed prior to further incubation. Catalase expression was induced with 5 ng mL^−1^ anhydrotetracycline after a total incubation time of 43 h. After 48 h incubation was stopped, nLRs were washed several times with nLR wash buffer and stored at 4 °C in the buffer until separation.

#### Preparation of nLRs with B_2_ producers/biosensors for buoyancy separation

B_2_ producer strains harbouring the different B_2_ overproduction plasmids were grown from frozen stocks in 5 mL LB-Miller broth (supplemented with 50 µg mL^−1^ kanamycin) in a pre-culture until mid-exponential phase (OD_600_ = 0.6 to 1). The biosensor was directly inoculated from a fresh vial of a B_2_ limited frozen stock (see above). Both strains were co-encapsulated in nLRs and incubated (100 g L^−1^ wet nLRs) at 37 °C on an orbital shaker (200 rpm, 25 mm amplitude) in LB-ΔRib broth supplemented with 20 µg mL^−1^ kanamycin. At this stage of incubation, only the B_2_ producer strains can form microcolonies within the nLRs (due to their endogenous B_2_ synthesis) while the B_2_ auxotrophic biosensor does not grow. After incubation for 7 h, B_2_ production (encoded on the plasmid) was induced with 2.5 ng mL^−1^ anhydrotetracycline and expression of the Cat-GFP marker was induced with 20 µM isopropylthiogalactoside (IPTG). The nLRs were incubated for an additional hour and then removed from the medium by sieving. Next, the nLRs were dispersed in paraffin oil (100 g L^−1^ wet nLRs) supplemented with 20 g L^−1^ Abil EM90 (Evonik Goldschmidt, Essen, Germany) and 1 g L^−1^ Tween 20 as emulsifiers and using a paddle mixer operated at 1,000 rpm for 30 s. The emulsion was then incubated in 50 mL aliquots in polystyrene containers with screw caps (TP51-011, Gosselin, Hazebrouck, France) at 37 °C on an orbital shaker (200 rpm, 25 mm amplitude). After 22 h, the nLRs were recovered by decanting the oil and washing the remaining slurry (containing the nLRs) several times with nLR wash buffer until all the oil was removed. The nLRs were then stored at 4 °C in the buffer until further use.

### Buoyancy separation

#### Separation in small scale

For initial characterization of the buoyancy properties, experiments were performed in small scale (10 to 25 nLRs at a time) and the separation was performed and evaluated directly under the microscope. After incubation and isolation of the nLRs from growth medium, monoclonal nLRs displaying the desired phenotype (e.g. a certain microcolony size or catalase activity) were distributed over rectangular Nunc OmniTray plates using large-particle flow cytometry. With each sorted nLR, approx. 4 µL of sheath-fluid (10 mM CaCl_2_ in water) was spotted on the dish. Depending on the experiment, typically 10 to 25 nLRs were spotted in one place and therefore an aqueous volume of 40 to 100 µL was accumulated per spot. Separation was initiated by quickly adding an approximately equal volume (40 to 100 µL) of diluted H_2_O_2_ (3.5% in nLR wash buffer) to each of the spots (final H_2_O_2_ concentration approx. 1.75%). The gas liberation process was followed by bright field microscopy and nLRs with positive buoyancy were counted in the focal plane at the top while nLRs with negative buoyancy were counted at the focal plane at the bottom of the droplet. For each condition, normally 5 to 10 spots were assayed in this way, adding to roughly 60 to 250 analysed microcolony-occupied nLRs per condition.

#### Separation in large scale

Separation of large numbers of nLRs was performed in a custom-made separator device inspired by the design of a fish trap (see Supplementary Fig. 8a). First, the isolated nLRs (up to 5 × 10^7^, suspended in approx. 500 mL of nLR wash buffer) were transferred through the outlet at the top into the sampler chamber of the device containing approx. 500 mL of nLR wash buffer (corresponding to about half the total volume of the device). Next, the mixture was allowed to stand until the nLRs had settled on the bottom of the sampler. Then, 200 mL of diluted H_2_O_2_ (2% in nLR wash buffer) was slowly pumped in from the feeder through the porous bottom of the sampler chamber. Note that the final concentration of H_2_O_2_ in the sampler was thus <2%. Within 10 to 20 seconds, positive nLRs (i.e. nLRs containing catalase expressing biosensors) started to float and readily rose to the top of the suspension. Due to the continuous supply of H_2_O_2_ to the system the liquid volume in the separator device slowly increased. This led to an outflow of the liquid over the upper rim of the inner tube of sampler into the collector, initially filled with 50 mL of nLR wash buffer. This liquid flow supports the removal of floating nLRs from the inner part of the device. Note that the nLRs are no longer exposed to high concentrations of H_2_O_2_ once they entered the collector filled with H_2_O_2_-free wash buffer. After approx. 60 s the process was completed and the recovered fraction was removed from the collector with a cell strainer (100 µm mesh-size, Becton Dickinson, Franklin Lakes, NJ), washed with an excess of nLR wash buffer and stored at 4 °C in the same buffer (see Supplementary Fig. 8d). For counting of nLRs, the isolated fractions were washed several times with nLR wash buffer to remove free cells in the supernatant of the nLRs and then analysed using large-particle flow cytometry. If required, large-particle flow cytometry was used to spot single nLRs onto Nunc OmniWell plates filled with 50 mL LB-Miller agar supplemented with the appropriate antibiotic(s). Plates were incubated until visible microcolonies were formed which were then genotyped by colony PCR and Sanger sequencing.

## Electronic supplementary material

Supplementary Information

Supplementary Video 1: Oxygen production and capturing in nanolitre reactors.

Supplementary Video 2: Demonstration model for buoyancy separation.

## References

[1] Taylor SV, Kast P, Hilvert D (2001). Investigating and engineering enzymes by genetic selection. Angew. Chemie Int. Ed..

[2] Yang G, Withers SG (2009). Ultrahigh-throughput FACS-based screening for directed enzyme evolution. ChemBioChem.

[3] Chen B (2016). High-throughput analysis and protein engineering using microcapillary arrays. Nat. Chem. Biol..

[4] Joensson HN, Andersson Svahn H (2012). Droplet microfluidics-a tool for single-cell analysis. Angew. Chemie Int. Ed..

[5] Yan C (2017). Real-time screening of biocatalysts in live bacterial colonies. J. Am. Chem. Soc..

[6] Walser M, Leibundgut RM, Pellaux R, Panke S, Held M (2008). Isolation of monoclonal microcarriers colonized by fluorescent *E*. *coli*. Cytom. Part A.

[7] Walser M (2009). Novel method for high-throughput colony PCR screening in nanoliter-reactors. Nucleic Acids Res..

[8] Meyer A (2015). Optimization of a whole-cell biocatalyst by employing genetically encoded product sensors inside nanolitre reactors. Nat. Chem..

[9] Roberts TM (2016). Identification and characterisation of a pH-stable GFP. Sci. Rep..

[10] Salsac, A.-V., Zhang, L. & Gherbezza, J.-M. Measurement of mechanical properties of alginate beads using ultrasound. *19eme Congr*. *Fr*. *Mec*. 1–6 (2009).

[11] Haas A, Brehm K, Kreft J, Goebel W (1991). Cloning, characterization, and expression in *Escherichia coli* of a gene encoding *Listeria seeligeri* catalase, a bacterial enzyme highly homologous to mammalian catalases. J. Bacteriol..

[12] Bienaimé C, Barbotin J-N, Nava-Saucedo J-E (2003). How to build an adapted and bioactive cell microenvironment? A chemical interaction study of the structure of Ca-alginate matrices and their repercussion on confined cells. J. Biomed. Mater. Res. Part A.

[13] Wang HH (2009). Programming cells by multiplex genome engineering and accelerated evolution. Nature.

[14] Jiang W, Bikard D, Cox D, Zhang F, Marraffini LA (2013). RNA-guided editing of bacterial genomes using CRISPR-Cas systems. Nat. Biotechnol..

[15] Salis HM, Mirsky EA, Voigt CA (2009). Automated design of synthetic ribosome binding sites to control protein expression. Nat. Biotechnol..

[16] Espah Borujeni A, Channarasappa AS, Salis HM (2014). Translation rate is controlled by coupled trade-offs between site accessibility, selective RNA unfolding and sliding at upstream standby sites. Nucleic Acids Res..

[17] Schwechheimer SK, Park EY, Revuelta JL, Becker J, Wittmann C (2016). Biotechnology of riboflavin. Appl. Microbiol. Biotechnol..

[18] Vogl C (2007). Characterization of riboflavin (vitamin B2) transport proteins from *Bacillus subtilis* and *Corynebacterium glutamicum*. J. Bacteriol..

[19] Wittmann A, Suess B (2012). Engineered riboswitches: expanding researchers’ toolbox with synthetic RNA regulators. FEBS Lett..

[20] Roth A, Breaker RR (2009). The structural and functional diversity of metabolite-binding riboswitches. Annu. Rev. Biochem..

[21] Zhang F, Carothers JM, Keasling JD (2012). Design of a dynamic sensor-regulator system for production of chemicals and fuels derived from fatty acids. Nat. Biotechnol..

[22] Taylor ND (2015). Engineering an allosteric transcription factor to respond to new ligands. Nat. Methods.

[23] Dietrich JA, McKee AE, Keasling JD (2010). High-throughput metabolic engineering: advances in small-molecule screening and selection. Annu. Rev. Biochem..

[24] Rogers JK, Taylor ND, Church GM (2016). Biosensor-based engineering of biosynthetic pathways. Curr. Opin. Biotechnol..

[25] Amstad E (2016). Robust scalable high throughput production of monodisperse drops. Lab Chip.

[26] Buffi N (2011). Development of a microfluidics biosensor for agarose-bead immobilized *Escherichia coli* bioreporter cells for arsenite detection in aqueous samples. Lab Chip.

[27] Fischlechner M (2014). Evolution of enzyme catalysts caged in biomimetic gel-shell beads. Nat. Chem..

[28] Duarte JM, Barbier I, Schaerli Y (2017). Bacterial Microcolonies in gel beads for high-throughput screening of libraries in synthetic biology. ACS Synth. Biol..

[29] Colin P-Y, Zinchenko A, Hollfelder F (2015). Enzyme engineering in biomimetic compartments. Curr. Opin. Struct. Biol..

[30] Neuenschwander M, Butz M, Heintz C, Kast P, Hilvert D (2007). A simple selection strategy for evolving highly efficient enzymes. Nat. Biotechnol..

[31] Ronda C, Pedersen LE, Sommer MOA, Nielsen AT (2016). CRMAGE: CRISPR optimized MAGE recombineering. Sci. Rep..

[32] Guo D (2015). Online high-throughput mutagenesis designer using scoring matrix of sequence-specific endonucleases. J. Integr. Bioinform..

[33] Link AJ, Phillips D, Church GM (1997). Methods for generating precise deletions and insertions in the genome of wild-type *Escherichia coli*: application to open reading frame characterization. J. Bacteriol..

