# Supplementary material for: Archimedes’ principle for characterisation of recombinant whole cell biocatalysts
Source: Sci Rep. 2018 Feb 14;8:3000. doi: 10.1038/s41598-018-20877-1 (PMC5813238; doi:10.1038/s41598-018-20877-1)
Supplement: Supplementary file 1 — Supplementary Information [file 41598_2018_20877_MOESM1_ESM.pdf]

Supplementary Information

Archimedes' principle for characterisation of recombinant whole cell biocatalysts

Steven Schmitt, Marcel Walser, Michael Rehmann, Sabine Oesterle, Sven Panke & Martin Held

Table of contents

Supplementary Notes **3**

Theoretical considerations for separations based on density shifts. 3

Potential of the Archimedes technology. 4

Supplementary Methods **6**

Chemicals and molecular biology. 6

Construction of catalase knock-outs. 7

Assay of *in vivo* catalase activity 8

Measurement of *E. coli* growth kinetics. 9

B_2 secretion by *E. coli*. 9

Quantification of B_2 secretion. 11

Fluorescent labelling of nLRs. 11

Fluorescence microscopy. 12

Large-particle flow cytometry. 13

Supplementary Tables **14**

Supplementary Table 1. DNA oligonucleotides used in this study. 14

Supplementary Table 2. *E. coli* strains used in this study. 15

Supplementary Table 3. Plasmids used in this study. 16

Supplementary Figures **17**

Supplementary Figure 1. O_2 release of *E. coli* MDS42 cells. 17

Supplementary Figure 2. Demonstration model for buoyancy separation. 18

Supplementary Figure 3. Recovery of nLR-embedded *E. coli* MDS42 cells. 19

Supplementary Figure 4. Assay types suitable for buoyancy separation. 20

Supplementary Figure 5. Growth rates of strains with *lacZ* RBS variants in liquid culture. 21

Supplementary Figure 6. Growth parameters of fast- and slow-growing *E. coli* in nLRs. 22

Supplementary Figure 7. Separation of fast- and slow-growing *E. coli*. 23

Supplementary Figure 8. Function principle of the separator device. 24

Supplementary Figure 9. B_2 biosynthesis in *E. coli*. 26
Supplementary Figure 10. B_2 production and sensing in nLRs. 27
Supplementary References 28

Supplementary Notes

Theoretical considerations for separations based on density shifts.

We aimed at using inducible density shifts as separation trigger for high-throughput screening and selection assays. Therefore, as a first step, we established a marker that allowed adjusting the density of the analysed object such that “positive” library variants decrease their density and their buoyancy is inverted from negative (sedimenting) to positive (ascending, i.e. become buoyant), which allows their recovery from the top of the cultivation liquid. We considered the release of O_2 from cells and the capturing of gas to reduce the density of the analysed object. To capture the gas released by the cells we employ nLRs, the formation of a gas-filled chamber within the nLRs would then result in an overall density shift of the reactor. Typically, nLRs consisting of 20 g L^{-1} alginate have a density[1] of approx. 1.1 g mL^{-1} and are suspended in an aqueous buffer of a density of approximately 1 g mL^{-1}. As an estimate, we targeted a density change of more than 20% to less than 0.9 g mL^{-1} in order to efficiently reverse the buoyancy of the nLR from negative to positive. Using nLRs with a diameter of 460 µm as an example, an embedded colony would have to release approx. 10 nL gas (or 450 pmol) within a timeframe that allows for capturing of the gas within the matrix of the nLR (seconds to few minutes). If the release was too slow, the “gas” would simply remain dissolved in the water, diffuse out of the nLR and no bubble would form. Assuming a maximum colony size of 100,000 cells $colony^{-1}$ within an nLR, such amounts cannot be provided by metabolic activity of the cells. For example, the release of CO_2 by *E. coli* due to glucose catabolism results in a cellular release of approx. 2 fL CO_2 $cell^{-1}$ min^{-1} (BNID 107919[2,3]). Even if all released CO_2 were gaseous and subsequently captured within the nLR, a production time of 45 min would be required to fill the reactor with sufficient amounts of CO_2, which is impractical. We, therefore, considered a dedicated enzymatic reaction such as catalysed by catalases, which catalyse the decomposition of hydrogen peroxide to water and oxygen[4]

(EC 1.11.1.6), or by carbonic anhydrases, which catalyse the interconversion of carbon dioxide and water to bicarbonate and protons[5] (EC 4.2.1.1), and that - at least in principle - allow for high gas release rates. We selected catalases for further characterization as they allow for simple triggering of O_2 release by adding H_2O_2 and we could show that overexpression of such an enzyme can result in a production of up to 9 pL O_2 $cell^{-1}$ min^{-1} (see Supplementary Fig. 1). This was sufficient to generate the required density shift in less than 1 s.

Potential of the Archimedes technology.

Scaling of the analysis and separation step:

Buoyancy separation takes advantage of nLRs which serve as compartments for microcolony growth and for synthesis and retention of gaseous O_2. For the Archimedes technology, analysis and separation are combined in a single step which is performed batch wise within a few seconds. To this end, analysis and separation rates mainly depend on the size of the batch and therefore on the capacity of the separator device and the size of the nLRs.

The separator device used in this study was constructed by S.S. and M.H. and manufactured by the ETH workshop. The key parameters for its capacity are the diameter of the separation chamber and the thickness of the layer of nLRs-sediment prior to the addition of H_2O_2 and buoyancy separation (see table below; “demonstrated”). Based on a number of exploratory experiments performed by us in the course of the device development and our principal understanding of engineering, we argue that both the diameter of the separation chamber and the thickness of the layer can be easily increased further (table below; “optimized”). The throughput can also be further increased by reduction of the size of the nLRs.

Table. Scaling of the analysis and separation step.

Separator device capacity	Chamber diameter	Layer thickness	Diameter nLR 460 µm	230 µm	100 µm	50 µm	20 µm
**Demonstrated**	130 mm	30 mm	6×10^6 nLRs*	5×10^7 nLRs*	6×10^8 nLRs*	5×10^9 nLRs*	7×10^{10} nLRs*
**Optimized**	400 mm	100 mm	2×10^8 nLRs*	1×10^9 nLRs*	2×10^{10} nLRs*	1×10^{11} nLRs*	2×10^{12} nLRs*

* Per batch and estimating a close-packing of reactors of 74%

nLR size and production rates:

A plethora of well-established technologies allow rapid production of monodisperse nLRs over a vast range of diameters (see table below). The respective technologies can be subdivided into falling-droplet-based (droplets are generated in air and collected in an aqueous buffer) and emulsion-based (employing a water-immiscible liquid phase for nLR production) methods. The respective hardware usually relies on nozzles or junctions for the production of hydrogel microcompartments at frequencies between 500 and 5,000 per second. Most of these technologies are amenable to multiplexing[6,7], typically resulting in an increase of the nLR production rates by one to two orders of magnitude. If monodispersity is not mandatory, even faster technologies for production of microcompartments in bulk or at high frequencies (>>10,000 per second) can be applied. Examples are certain spraying protocols[8] or bulk emulsification processes[9].

Table. nLR size and production rates.

	Falling-droplet-based		Emulsion-based	
	**Laminar jet breakup**	**Air flow / spray**	**Microfluidic chips**	**Bulk**
Size distribution	Monodisperse	Polydisperse	Monodisperse	Polydisperse
Reactor diameter [µm]	200 - 1000	40 - 500	20 - 100	<100
Reactor volume [nL]	4 - 524	0.03 - 65	0.004 - 0.5	<0.5
Production throughput [kHz]	1 - 5	5 - 50	0.5 - 5	Bulk (minutes)
Multiplexing possible	Yes (>10)	Yes (>10)	Yes (>500)	NA
Typical batch size single nozzle*	10^7	10^8	10^7	$>>10^7$
Typical batch size multi nozzle*	10^8	10^9	10^9	NA
References	[6,10]	[8]	[7,11,12]	[9,13]

* Typical batch sizes are estimated based on a production time not exceeding 1 h.

Supplementary Methods

Chemicals and molecular biology.

Unless otherwise noted, chemicals were obtained from Sigma-Aldrich (St. Louis, MO). All cloning steps were performed in *E. coli* DH5α (see Supplementary Table 2 for a comprehensive list of strains) obtained from Thermo Fisher Scientific (Waltham, MA). Routine strain cultivations were performed in LB-Miller broth (Difco, Becton Dickinson, Franklin Lakes, NJ). LB-Miller depleted of B_2 (LB-ΔRib) was generated by photo-bleaching as follows: An aliquot of 400 mL of sterile LB-Miller broth (provided in a 500 mL Erlenmeyer flask) was incubated in a customized light chamber (radiation at 470 nm provided from 6x6 LED, operated at a power consumption of approx. 3.7 W for 48 h under magnetic stirring at 50 rpm). B_2 depletion was confirmed by the absence of growth of B_2 auxotrophic strains in a control aliquot removed from the medium. Chemically defined medium (CDM) contained in ddH_2O 50 mM MES, 9.0 mM potassium phosphate, 148.87 mM sodium chloride, 7.04 mM calcium chloride, 0.98 mM magnesium chloride, 20.21 µM manganese(II) chloride, 0.24 µM ammonium molybdate, 1.07 µM cobalt(II) sulphate, 1.20 µM copper(II) sulphate, 1.04 µM zinc sulphate, 20.12 µM iron(III) chloride, 9.69 µM (±)-α-lipoic acid, 2.10 µM D-pantothenic acid, 8.12 µM nicotinic acid, 4.91 µM pyridoxal hydrochloride, 4.86 µM pyridoxine hydrochloride, 2.96 µM thiamine hydrochloride, 0.41 µM biotin, 1.46 mM L-alanine, 1.40 mM L-arginine, 0.61 mM L-asparagine, 1.03 mM L-aspartic acid, 0.35 mM L-cysteine, 0.66 mM L-glutamic acid, 0.66 mM L-glutamine, 0.39 mM glycine, 0.16 mM L-histidine, 0.63 mM L-isoleucine, 0.89 mM L-leucine, 1.02 mM L-lysine, 0.26 mM L-methionine, 0.39 mM L-phenylalanine, 3.58 mM L-proline, 1.64 mM L-serine, 0.57 mM L-threonine, 0.18 mM L-tryptophan, 2.76 mM L-tyrosine, 0.73 mM L-valine and was adjusted to pH 7.0 with sodium hydroxide. CDM was either supplemented with 4 g L^{-1} D-glucose or 4 g L^{-1} D-lactose and the appropriate antibiotic(s) for plasmid maintenance.

If not noted otherwise, incubations of the cultures were performed at 37 °C (except for *E. coli* EcNR1 which was incubated at 30 °C) either in 14 mL polypropylene centrifugation tubes (Greiner, Kremsmünster, Austria) with aeration on a shaker (Kuhner, Birsfelden, Switzerland) operated at 200 rpm and 25 mm amplitude or in Nunc DeepWell 96-well plates (95040452, Thermo Fisher Scientific, Waltham, MA) on a shaker equipped with the Duetz-System (Kuhner, Birsfelden, Switzerland) operated at 300 rpm and 50 mm amplitude. For long-term storage of strains, the culture was supplemented with glycerol to a final volume fraction of 20% and frozen at -80 °C. Isolation of plasmid DNA from cells, the purification of PCR-products and the DNA extraction from gels were performed using kits from Zymo Research (Irvine, CA). Enzymes were obtained from NEB (Ipswich, MA), used together with the appropriate buffers and according to the manufacture's recommendations. Sanger sequencing was performed at Microsynth (Balgach, Switzerland) and GATC Biotech (Konstanz, Germany) using purified plasmids or unpurified PCR products, an appropriate primer (see Supplementary Table 1 for a comprehensive list of DNA oligonucleotides) and following the instructions supplied by the service provider. The nLRs were prepared from sodium alginate (20 g L^{-1} in ddH_2O, sterile-filtered, see also below), gelled in nLR hardening buffer (100 mM $CaCl_2$, 1 mM Tris-HCl, pH 7) and washed/stored in nLR wash buffer (10 mM $CaCl_2$, 1 mM Tris-HCl, pH 7).

Construction of catalase knock-outs.

We used the single catalase knock-outs of *E. coli* BW25113, BW25113 Δ*katE*::*kan* and Δ*katG*::*kan* from the Keio collection[14] as template for the construction of a double knock-out strain lacking both, *katE* and *katG*. First, the correct genotypes of the strains were verified by colony PCRs of the genomic regions containing *katE* (primers ID 2 and ID 3) and *katG* (primers ID 4 and ID 5) followed by Sanger sequencing (primers ID 2 or ID 4) of the PCR product. Then, the Δ*katG*::*kan* knock-out was phage-transduced[15] into BW25113 Δ*katE*, obtained from

BW25113 Δ*katE*::*kan* by removal of the kanamycin resistance gene as described elsewhere[16]. Knock-outs were verified by genomic colony PCR and Sanger sequencing as described above.

Assay of *in vivo* catalase activity.

Catalase activity was assayed spectrophotometrically (240 nm) via the H_2O_2 turnover[17]. Briefly, strains were grown in 5 mL LB-Miller broth, if required supplemented with 20 µg mL^{-1} chloramphenicol, until an OD_{600} of approx. 0.6 was reached. Catalase expression from pCat was induced by adding the desired concentration (0 to 200 ng mL^{-1}) of anhydrotetracycline and incubation was continued overnight. By the next day, cultures typically reached an OD of 3.5 and the suspensions were diluted to 3.4 x 10^7 cells mL^{-1} (OD_{600} = approx. 0.07) in TBS buffer (154 mM sodium chloride, 10 mM Tris-HCl, pH 7). For the assay, 0.5 mL of the diluted cell suspension was filled into a quartz cuvette and the assay was started by adding 0.5 mL of 120 mM H_2O_2 in TBS buffer. The suspension was quickly mixed by inverting the cuvette and the UV/Vis signals were recorded immediately afterwards on a Lambda 25 spectrophotometer (Perkin Elmer, Waltham, MA, 1 nm BP, 1 Hz) for 180 s. All measurements were performed at room temperature. The H_2O_2 concentration at each time point was calculated by comparing the absorbance in the sample to the absorbance of a H_2O_2 standard measured prior the experiment (0 to 60 mM). The depletion of H_2O_2 for each sample was plotted and the initial (linear) part of the depletion curve was used for calculation of the H_2O_2 depletion rate. We used *E. coli* BW25113 Δ*katE*::*kan* Δ*katG*, grown under the same conditions, to correct for catalase independent H_2O_2 depletion (which is mainly due to the function of NADH peroxidases[18]) and usually amounted to approx. 15 fmol cell^{-1} min^{-1}. The depletion, solely based on catalase activity, was then used to deduce the O_2 release of samples according to 2 H_2O_2 = 2 H_2O + O_2, and the O_2 release of a single *E. coli* cell was calculated by division of that value by the number of cells

(measured by OD_{600}, with 1 OD_{600} equivalent to 5 x 10^8 cells mL^{-1}, as determined by CFUs on LB-Miller agar) in the suspension. All measurements were done in triplicate.

Measurement of *E. coli* growth kinetics.

To determine growth parameters of the *E. coli* EcNR1 strains, pre-cultures derived from single clones were grown in 800 µL LB-Miller broth supplemented with 20 µg mL^{-1} chloramphenicol and 50 µg mL^{-1} kanamycin in a DeepWell plate at 30 °C. Next, 5 µL of the pre-culture were added to 95 µL CDM, supplemented with 20 µg mL^{-1} chloramphenicol and 50 µg mL^{-1} kanamycin and either 4 g L^{-1} D-glucose or 4 g L^{-1} D-lactose in a Nunc MicroWell 96-well plate with a clear bottom (167008, Thermo Fisher Scientific, Waltham, MA). The plate was closed with the lid (supplied with the plate) and growth kinetics were recorded on an Infinite M200 PRO platereader (Tecan, Männedorf, Switzerland) operated at 30 °C with orbital shaking at a 4 mm amplitude. Measurements were performed every 15 min until all strains reached the stationary-phase. Data were exported as comma-separated file, imported into a custom-made R-script fitting the logistic equation ($N_t = K (1+((K-N_0) N_0^{-1} e^{-\mu t})^{-1})$) to the data using the Growthcurver package and employing a non-linear least-squares Levenberg-Marquardt algorithm[19] to deduce the specific growth rate µ. All measurements were done in quadruplicate.

B_2 secretion by *E. coli*.

The biosynthesis operon for the overproduction of B_2 in *E. coli* was assembled after PCR amplification of the B_2 synthesis genes (*ribABCDE*) from the genome of *E. coli* BW25113 using primers ID 17 to ID 26 and their assembly in a synthetic operon. All of the isolated genes no longer contained any of the known regulatory elements. Each fragment was provided with a HindIII restriction site at the 5' terminus and an NheI site followed by an EcoRI site at the 3' terminus (integrated with the primers used for amplification). Gene assembly to the operon was

performed similarly to the BioBrick standard protocol[20]. Briefly, each amplicon was digested with HindIII and EcoRI and separately ligated into plasmid pAB92 linearized with the same enzymes. The plasmid contained a ribosome binding site (RBS, GAAGGAGAT, located at position -17 to -9, relative to the start codon) and the P_{tet} promoter, providing a set of 5 basic plasmids, one for each of the *rib* genes. Next, the plasmid containing the biosynthesis gene considered to be the first in the operon was linearized with NheI and EcoRI and the second gene of the operon was excised (together with the RBS) from its basic plasmid with XbaI and EcoRI. The second gene was then ligated into the linearized plasmid containing the first gene, destroying the XbaI and NheI sites in the process and resulting in a position downstream of the first gene. This process was repeated until the operon was completed. Next, the operon was transferred into the backbone pB2_empty for the final production plasmids. Plasmid pB2_empty was derived from pSEVA261, additionally equipped with a cassette for chloramphenicol resistance (thus providing a kanamycin and chloramphenicol double-resistance). For this, the chloramphenicol resistance cassette was amplified from pAct3 using primers ID 31 and ID 32 fusing the blunt-end restriction-sites EcoRV and PshAI to the fragment. The amplicon was digested with both enzymes and the fragment was ligated into the pSEVA261 plasmid, linearized with PshAI. Ligations resulted in the plasmid pB2_empty carrying both, the kanamycin and chloramphenicol resistance cassette and was verified using colony PCR and primers ID 32 and ID 33. The complete B_2 biosynthesis operon together with its regulatory elements was then excised from pAB92 by cleavage with SpeI and EcoRI, cloned into pB2_empty linearized with the same enzymes, which yielded the B_2 production plasmid pB2_*ribDBECA* and pB2_*ribBECA*, depending on the transferred operon. The promoter-less operon variant pB2_$ribDBECA_{PL}$ was generated by restriction digest of the full production plasmid pB2_*ribDBECA* with SpeI and XbaI, gel purification and recircularization of the plasmid, thus removing the P_{tet} promoter and its

repressor gene *tetR*. All assemblies were verified using Sanger sequencing with primers ID 1 and ID 27 to ID 30. All plasmids, including a negative control plasmid (plasmid pB2_empty) were used to transform the *E. coli* strain BW23474, yielding the final B_2 production (and control) strains.

Quantification of B_2 secretion.

The amount of secreted B_2 was quantified by UV/Vis spectroscopy. Strains were grown overnight at 37 °C in 800 µL LB-Miller broth, supplemented with 20 µg mL^{-1} kanamycin in Nunc DeepWell 96-well plates. From the stationary-phase culture, a new plate with 800 µL LB-ΔRib broth supplemented with 20 µg mL^{-1} kanamycin and 2.5 ng mL^{-1} anhydrotetracycline was inoculated (1:100) and incubated for 20 h. Cell density was measured by diluting the culture 1:10 in ddH_2O, adding 200 µL of the diluted culture into a clear-bottom Nunc MicroWell 96-well plate and measuring the optical density at 600 nm using an Infinite M200 PRO platereader. The remaining production culture was centrifuged (3,200 g, 15 min) and 200 µL of the cell-free supernatant was transferred to a fresh 96-well plate. The B_2 amount secreted by the culture was quantified by measuring the absorbance at 444 nm against identically treated supernatant from the non-B_2-producing *E. coli* strain BW23474 [pB2_empty]. The corrected absorbance values for each of the samples were compared to those from a B_2 standard (250 to 0.5 µM, $\log_2$ dilution in 154 mM aqueous NaCl) to deduce the B_2 concentration in the sample. All measurements were done in triplicate.

Fluorescent labelling of nLRs.

If required, nLRs were labelled with ROX silica particles (red fluorescent; excitation-peak: 578 nm; emission-peak: 604 nm) or alginate-bound fluorescein (green fluorescent; excitation-peak: 490 nm; emission-peak: 525 nm) allowing their identification by large-particle flow

cytometry or fluorescence microscopy. ROX silica particles were prepared using NHS-coupling chemistry[21]. Briefly, aliquots comprised of 500 µL of 3-aminopropyl-functionalized silica nanoparticles (<100 nm particle size, 3 g L^{-1} in ethanol) and 50 µL of the amine-reactive red dye 5(6)-carboxy-X-rhodamine-N-hydroxysuccinimide ester (5(6)-ROX SE (20 mM in DMSO, Chemodex, St. Gallen, Switzerland)) were mixed in 1.5 mL centrifugation tubes, incubated on a tube rotator (4 hours, room temperature) and afterwards stored at 4 °C. Prior to usage, the particles were precipitated by the addition of 200 µL of ddH_2O, pelleted (14,000 g, 10 min), washed two times with 500 µL of ddH_2O, resuspended in 500 µL ddH_2O using a lab-scale ultra-sonication bath (20 min) and added to the alginate prior to nLR synthesis to a final volume-fraction of 0.5%. Fluorescein-labelled alginate solution was prepared from 5-aminofluorescein and alginate using EDC/NHS-coupling chemistry as described elsewhere[22]. The resulting labelled alginate was used to prepare a stock solution of 5 g L^{-1} fluorescein-alginate in ddH_2O, which was sterile-filtered and stored at 4 °C until usage. For nLR labelling, the stock solution was added to the alginate prior to nLR synthesis at a final volume-fraction of 0.5%.

Fluorescence microscopy.

Microscopic analysis of nLRs was carried out with an inverted fluorescence microscope Axio Observer II equipped with an AxioCam MR3 camera (Carl Zeiss Microscopy, Jena, Germany) either using bright-field or epifluorescence with filter cubes (for GFP, SYTO 9 or fluorescein: excitation BP 470/40 nm, beam splitter DM 495 nm, emission BP 525/50 nm; for ROX: excitation BP 565/30 nm, beam splitter DM 585 nm, emission BP 620/60 nm). Images were taken with 25x, 50x, 100x or 400x magnification using the AxioVision software version 4.8.2 SP3. If bright-field and epifluorescence was recorded from the same object, images were processed and stored as overlays of fluorescent images and bright-field images. Image processing and analysis was done using Fiji software[23,24].

Large-particle flow cytometry.

For analysis and sorting of nLRs, large-particle flow cytometry (BioSorter, Union Biometrica, Holliston, MA) was employed. The device was operated with 10 mM $CaCl_2$ in water as sheath-fluid. Analysis was done using light-extinction at 488 nm as a trigger signal and recording data for time-of-flight (TOF, as a relative estimate of the particle size), extinction at 488 nm, green fluorescence (for GFP, SYTO 9 or fluorescein, excitation laser 488 nm, beam splitter DM 562, emission filter BP 510/23 nm) and red fluorescence (for ROX, excitation laser 561 nm, TR mirror, emission filter BP 615/24 nm). Signal analysis and selection of subpopulations were done using the Flowpilot software. Data analysis was performed using FlowJo software version 10.1 (FlowJo LLC, Ashland, OR). If required, nLRs were sorted bulk into 50 mL centrifugation tubes or arrayed on Nunc OmniTray plates (140156, Thermo Fisher Scientific, Waltham, MA), filled with 50 mL LB-Miller agar, supplemented with the appropriate antibiotic(s).

Supplementary Tables

Supplementary Table 1. DNA oligonucleotides used in this study.

ID	Name	Sequence (5' → 3')
1	Ptet_fw	ACCACTCCCTATCAGTGATA
2	katE_fw	CTTGTTTTCTCCCTCATTAC
3	katE_rv	TCCAGATAAGTGTGAGCAC
4	katG_fw	CTGTAGTTTAGCCGATTTAG
5	katG_rv	GCAATATATTGAATTTGCAC
6	CRISPR_lacZ_fw[a]	[P]AAACTGGAATTGTGAGCGGATAACAATTTCACACG
7	CRISPR_lacZ_rv[a]	[P]AAAACGTGTGAAATTGTTATCCGCTCACAATTCCA
8	MAGE_lacZ_pool[b,c]	T*G*C*T*TCCGGCTCGTATGTTGTGTGGAATTGTGAGCGGATAACAATTTC**ACACAG**GAAACAGCTATGACCATGATTACGGATTCACTGGCC
9	lacZ_fw	GTTGGCCGATTCATTAATG
10	lacZ_rv	GGACGACGACAGTATC
11	ribC_fw	CTATTATCTGAATGCCTGTGCAG
12	ribC_rv	TAGCGAGGATTTATTAATTCTCCAG
13	cat_NsiI_fw	AGCCGCATGCATACCGATAGAAGAAACTTAAC
14	cat_NsiI_rv	TCGGCGATGCATTTTTAAATCTATCACATGTT
15	pRSG_fw	GAGTTCGGCATGGGGTCAGGTGGG
16	pRSG_rv	GCGCCGACATCATAACGGTTCTGGC
17	ribA_HindIII _fw	CGTAGCAAGCTTATGCAGCTTAAACGTGTGGC
18	ribA_EcoRI_NheI_rv	ATAAGCGAATTCAACTACGCTAGCTTATTATTTGTTCAGCAAATGGC
19	ribB_HindIII _fw	CGTAGCAAGCTTATGAATCAGACGCTACTTTCCTCTTTTG
20	ribB_EcoRI_NheI_rv	ATAAGCGAATTCAACTACGCTAGCTTATCAGCTGGCTTTACGCTCAT
21	ribC_HindIII _fw	CGTAGCAAGCTTATGTTTACGGGGATTGTACAGGGC
22	ribC_EcoRI_NheI_rv	ATAAGCGAATTCAACTACGCTAGCTTATCAGGCTTCTGTGCCTGGTT
23	ribD_HindIII _fw	CGTAGCAAGCTTGTGCAGGACGAGTATTACATGG
24	ribD_EcoRI_NheI_rv	ATAAGCGAATTCAACTACGCTAGCTTATCATGCACCCACTAAATGCA
25	ribE_HindIII_fw	CGTAGCAAGCTTATGAACATTATTGAAGCTAACGTTGC
26	ribE_EcoRI_NheI_rv	ATAAGCGAATTCAACTACGCTAGCTTATCAGGCCTTGATGGCTTTCA
27	Operon_ribD_fw	CCTTGCTGATTCCAGAGCAT
28	Operon_ribB_fw	TGAGCGTAAAGCCAGCTGAT
29	pSEVA_t0	GGGGACCCCTGGATTCTCAC
30	pSEVA_t1	TACTCAGGAGAGCGTTCACC
31	CmR_EcoRV_fw	GCTGGATATCTTGGCGAAAATGAGACGTTG
32	CmR_PshAI_rv	TAATGACAAAAGTCAAATTACGCCCCGCCC

33	CmR_check_fw	TAATCCGGATATGAACAAACTGC

[a] Phosphorylated oligonucleotides are indicated with a [P] at the site of phosphorylation.
[b] Phosphorothioated bases are indicated with an asterisk after the base.
[c] See Supplementary Fig. 5 for RBS sequences, the *wt* RBS is shown in bold
Underlined sequences indicate restriction sites.

Supplementary Table 2. *E. coli* strains used in this study.

ID	Name	Genotype	Reference
1	DH5α	F⁻ Φ80*lacZ*ΔM15 Δ(*lacZYA-argF*)U169 *recA1 endA1* hsdR17(r_K^-, m_K^+) *phoA* supE44 λ⁻ *thi-1 gyrA96 relA1*	25
2	MDS42	based on MG1655, multiple deletion strain	26
3	BW25113	F⁻ Δ(*araD-araB*)567 Δ*lacZ4787*::*rrnB-3* LAM⁻ *rph-1* Δ(*rhaD-rhaB*)568 hsdR514	16
4	BW25113 Δ*katE*::*kan*	as BW25113 but Δ*katE*::*kan* (ECK1730)	14
5	BW25113 Δ*katG*::*kan*	as BW25113 but Δ*katG*::*kan* (ECK3934)	14
6	BW25113 Δ*katE*::*kan* Δ*katG*	as BW25113 but Δ*katE*::*kan* Δ*katG*	this work
7	EcNR1	as MG1655 but Δ(*ybhB-bioAB*)::[*λcI857* N(*cro-ea59*)::*tetR-bla*]	27
8	BW25113 Δ*ribC*::*ribM*	as BW25113 but with integration of *ribM* (incl. RBS) into the locus of *ribC*, RibM expression controlled by the *ribC* promoter, B_2 auxotrophic phenotype	this work
9	BW23474	F⁻ Δ(*argF-lac*)169 Δ*uidA4*::*pir-116 recA1* rpoS396(Am) *endA9*(del-ins)::FRT *rph-1* hsdR514 *rob-1* creC510	28

Supplementary Table 3. Plasmids used in this study.

ID	Name	Features	Reference
1	pAHA1	contains catalase gene from *L. seeligeri*	[29]
2	pAct3	p15A origin of replication, chloramphenicol resistance, P_{tac} promoter	[30]
3	pCat	as pAct3, but contains the catalase gene from *L. seeligeri* (from pAHA1) under control of the P_{tet} regulatory system	this work
4	pCP20	temperature sensitive origin of replication, chloramphenicol and ampicillin resistance, contains the gene for the FLP recombinase	[16,31]
5	pCRISPR	pBR322 origin of replication, kanamycin resistance, for expression of gRNA	[32], Addgene #42875
6	pCRISPR_*lacZ*	as pCRISPR, but contains the gene encoding for the gRNA sequence targeting the *lacZ* RBS-sequence of *E. coli* EcNR1	
7	pCas9	p15A origin of replication, chloramphenicol resistance, for expression of the *cas9* gene	[32], Addgene #42876
8	pKO3_*ribM*	pSC101ts origin of replication, temperature sensitive, chloramphenicol resistance, *ribC*::*ribM* construct for homologous recombination, *sacB* expression for counter selection on sucrose	[33,34]
9	pRSG_2A1	pBR322 origin of replication, ampicillin resistance, expression of *sfgfp* under transcriptional control of the P_{tac} promoter and translational control of the apatzyme 2A1	[34]
10	pSEVA281	pBR322 origin of replication, kanamycin resistance	[35]
11	pSense_*cat-gfp*	as pSEVA281, but expression of the *cat-gfp* fusion under transcriptional control of the P_{tac} promoter and translational control of the apatzyme 2A1	this work
12	pAB92	pBR322 origin of replication, ampicillin resistance, P_{tet}/T7 fusion promoter	[36]
13	pSEVA261	p15A origin of replication, kanamycin resistance	[35]
14	pB2_empty	as pSEVA261 but contains additionally the chloramphenicol resistance-cassette from pAct3	this work
15	pB2_*ribDBECA*	as pB2, but expression of the *ribDBECA* operon under control of the P_{tet}/T7 promoter	this work
16	pB2_*ribDBECA*$_{PL}$	as pB2_ribDBECA, but without P_{tet}/T7 fusion promoter and *tetR*	this work
17	pB2_*ribBECA*	as pB2, but expression of the *ribBECA* operon under control of the P_{tet}/T7 promoter	this work

Supplementary Figures

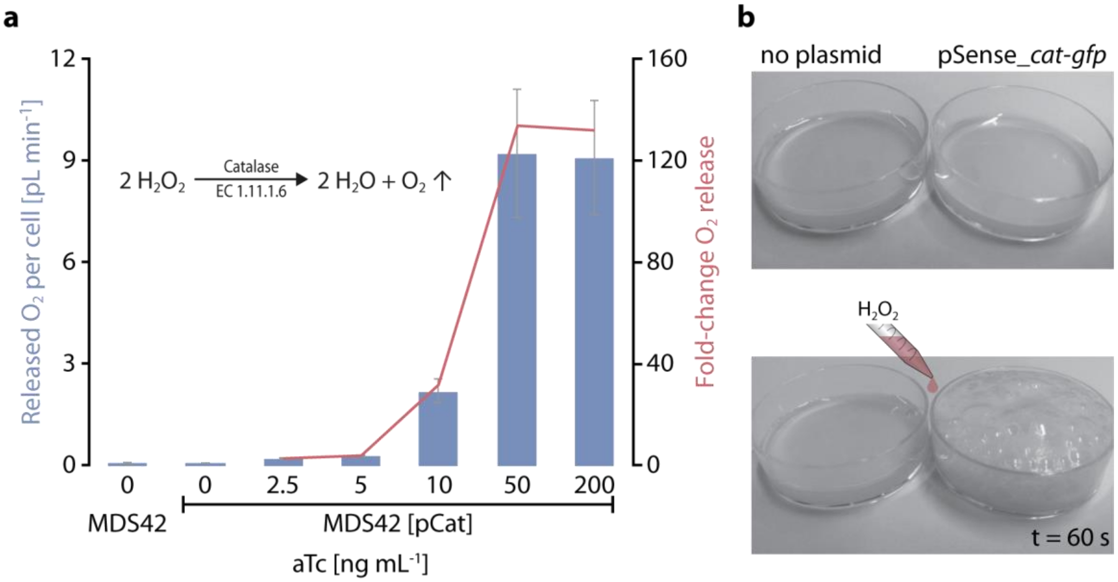

Supplementary Figure 1. O_2 release of *E. coli* MDS42 cells.

E. coli MDS42 [pCat] expresses a catalase gene from *L. seeligeri* via the anhydrotetracycline (aTc) inducible P_{tet} promotor. (a) H_2O_2 was added to freshly harvested cells and the catalase-catalysed O_2 production rate was calculated from the spectrophotometric measurement of the total H_2O_2 consumption rate reduced by the housekeeping H_2O_2 consumption rate determined from the catalase-free *E. coli* control strain (BW25113 Δ*katE*::*kan* Δ*katG*). The housekeeping rate was approx. 15 fmol cell^{-1} min^{-1}. Note that measurements were performed at low cell densities, and under these conditions O_2 was not lost to the gas phase, so measurements were monophasic. We observed an O_2 release between 69 fL cell^{-1} min^{-1} (approx. 3 fmol cell^{-1} min^{-1} for uninduced cells) and 9.2 pL cell^{-1} min^{-1} (approx. 410 fmol cell^{-1} min^{-1} for cells induced with 50 ng mL^{-1} aTc). The fold-change in O_2 release is calculated based on cells without induction. Data shown as mean ± SD, n=3. (b) O_2 release of strain *E. coli* BW25113 Δ*ribC*::*ribM* compared to the same strain overexpressing the catalase-GFP fusion from plasmid pSense_*cat-gfp*. Both strains were grown in LB-Miller (37 °C, overnight) supplemented with 20 µM B_2, 20 µg mL^{-1} kanamycin and 20 µM IPTG to an optical density OD_{600} of approx. 4.4. Next 4 mL of each of the strains were added to petri dishes (35 mm diameter, Sarstedt, Nümbrecht, Germany) and supplemented with 1.75% H_2O_2. After 60 s, images were taken. While there is barely any gas-development from *E. coli* BW25113 Δ*ribC*::*ribM* incubated in diluted H_2O_2 (left petri dish), strong foam-formation from the strain overexpressing the catalase-GFP protein indicates the release of massive amounts of O_2 (right petri dish).

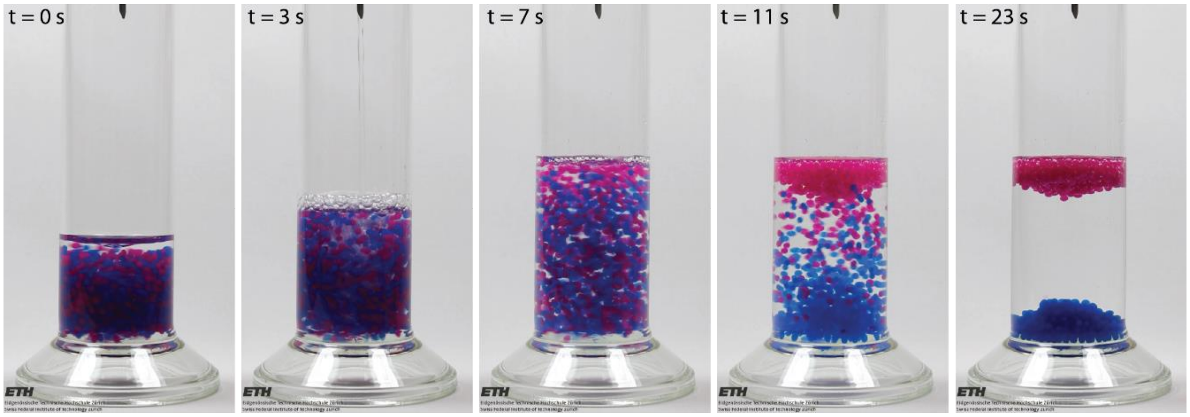

Supplementary Figure 2. Demonstration model for buoyancy separation.

Oversized demonstrator reactors (diameter approx. 2 mm, coloured with ink-particles) harbouring *E. coli* MDS42 [pCat] overexpressing the catalase gene (red) are separated via positive buoyancy from a background of empty carriers (blue). Note that the reactors were overpopulated (approx. 20 colonies per reactor) to ensure that each reactor contains a sufficient number of cells for separation. The approx. colony diameter after incubation was 50 µm, corresponding to approx. 65,000 cells colony^{-1}. At the beginning of the experiment both populations are mixed and dispersed in nLR wash buffer. After addition of an approximately equal volume of a solution containing 3.5% H_2O_2 in nLR wash buffer (finished at t = 7 s), the density of the positive gel carriers is decreased due to a displacement of water inside the nLR by O_2. This leads to an upward buoyancy force and the carriers start to ascend to the top from where they can be easily siphoned off. The snapshots are taken from Supplementary Video 2.

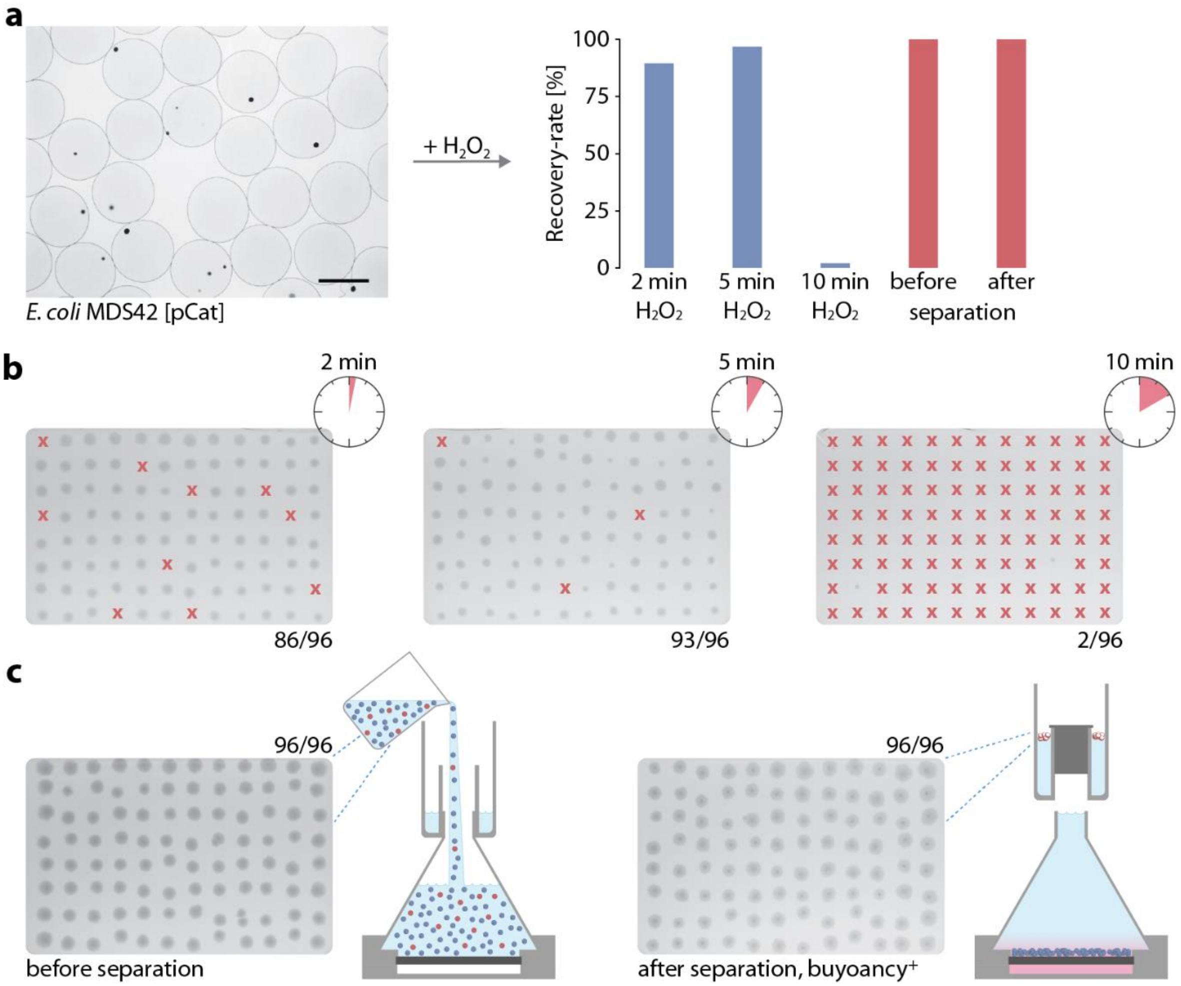

Supplementary Figure 3. Recovery of nLR-embedded *E. coli* MDS42 cells.

(a) Recovery of nLR-embedded *E. coli* MDS42 [pCat] colonies, grown for 15 h at 30 °C and treated with 2% H_2O_2 over different incubation times (blue bars, see also b, n=96) or separated in the separator device (red bars, see also c, n=96). Scale bar: 400 µm. (b) Colony-recovery of *E. coli* MDS42 [pCat] (catalase marker expression not induced), treated with H_2O_2 and incubated for different times. Shown are agar plates after spotting of H_2O_2-treated nLRs and outgrowth of nLR-embedded microcolonies to colonies. Note that a red "x" indicates an nLR spotted but no outgrowth (i.e. none of the cells within the microcolony survived the treatment). (c) Outgrowth of *E. coli* MDS42 [pCat] (induced with 10 ng mL^{-1} aTc for expression of the catalase marker gene) before and after separation in the separator device.

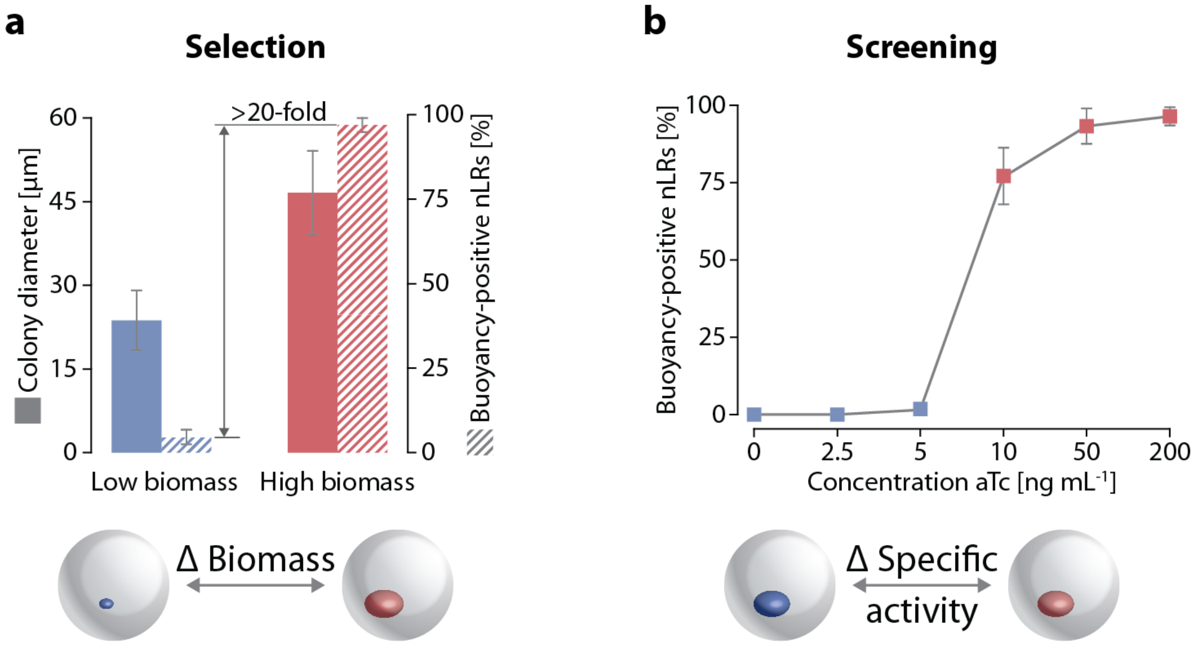

Supplementary Figure 4. Assay types suitable for buoyancy separation.

Density differences in nLRs can be triggered in two ways: (a) Variation of the biomass levels at a comparable cell-specific catalase activity (selection scenario). In this example, nLRs containing large colonies (average diameter of 46 µm, approx. 50,000 cells) are separated from nLRs harbouring only small colonies (average diameter of 23.5 µm, approx. 7,000 cells) while catalase expression was induced with 5 ng mL^{-1} anhydrotetracycline (aTc). Buoyancy separation under the microscope rendered 100 of 102 (98.0%) nLRs containing large colonies buoyancy-positive but only 5 of 114 (4.4%) filled with small ones. Data shown as mean ± SD, n=5 with 17 to 25 nLRs per experiment. (b) Alternatively, different catalase expression levels within nLRs containing similar cell numbers (average diameter of 27 µm, approx. 10,000 cells) can be employed (screening scenario). Here, increasing amounts of catalase activity per cell (indicated as increasing amounts of inducer concentration) allow for increased fractions of buoyancy-positive nLRs. Low catalase levels (expression induced with 0, 2.5 and 5 ng mL^{-1} aTc, leading to 0.07, 0.2 and 0.3 pL O_2 min^{-1} $cell^{-1}$, see also Supplementary Fig. 1), resulted in 0 of 69 (0%), 0 of 108 (0%) and 2 of 108 (1.9%) of buoyancy-positive nLRs whereas high catalase levels (expression induced with 50, 200 ng mL^{-1} aTc, resulting in 9.2 and 9.1 pL O_2 min^{-1} $cell^{-1}$) resulted in 104 of 111 (93.5%) and 80 of 83 (96.7%) floating nLRs. Data shown as mean ± SD, n=3 to 5 with 17 to 25 nLRs per experiment.

a

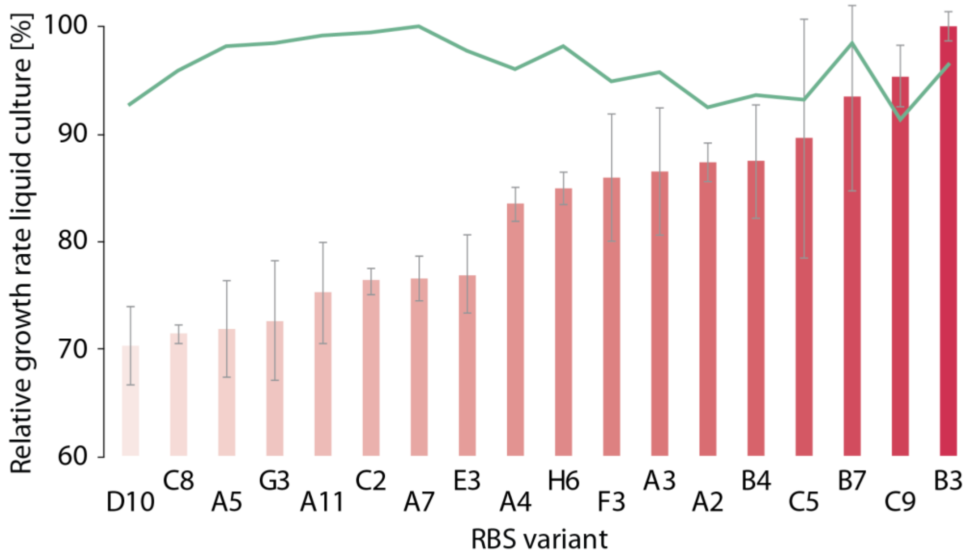

b

ID	RBS sequence	ID	RBS sequence	ID	RBS sequence	ID	RBS sequence
A2	TGGGGA	A3	GTGGGA	A4	TTGGGA	A5	TAGGGT
A7	TGGGGT	A11	CTGGGT	B3	TAGGGA	B4	CGGGGA
B7	GAGGGT	C2	CTGGGA	C5	CAGGGA	C8	TTGGGT
C9	GGGGGT	D10	GTGGGT	E3	GGGGGA	F3	GAGGGA
G3	CGGGGT	H6	CAGGGT	*wt*	ACACAG		

Supplementary Figure 5. Growth rates of strains with *lacZ* RBS variants in liquid culture.

(a) Specific growth rates of a collection of 18 different variants of *E. coli* strain EcNR1 carrying a modified RBS in front of the genomic *lacZ* gene, relative to the growth rate of the same strain carrying a wild type RBS sequence. The strains were grown in liquid culture in CDM and the relative growth rates of the strain grown with 4 g L^{-1} D-lactose (red bars) and 4 g L^{-1} D-glucose (green line) as carbon-source were calculated. Data shown as mean ± SD, n=4. (b) Table of RBS sequences (5’ → 3’) used in this study.

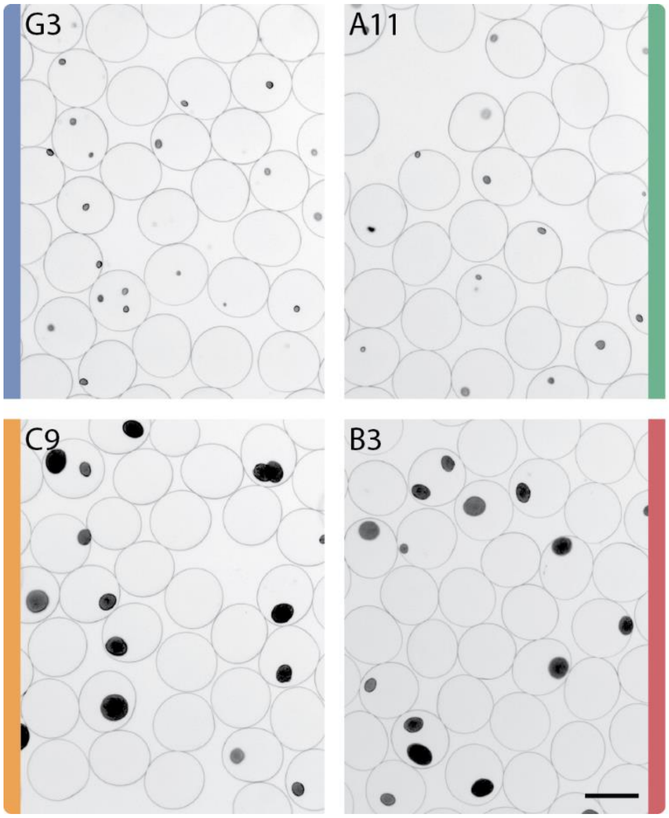

RBS variant	G3	A11	C9	B3
Colony diameter [µm] (SD)	20 (±4)	23 (±4)	57 (±15)	60 (±12)
Cell number per colony	~4k	~6k	~100k	~110k
(Relative) specific growth rate, colony [h^{-1}] ([%])	0.14 (72)	0.15 (75)	0.19 (99)	0.19 (100)
(Relative) specific growth rate, liquid [h^{-1}] ([%])	0.13 (72)	0.13 (75)	0.17 (95)	0.18 (100)

Supplementary Figure 6. Growth parameters of fast- and slow-growing *E. coli* in nLRs.

Four different variants of *E. coli* strain EcNR1_XX [pCRISPR_*lacZ* + pCat] with modulated RBSs for the *lacZ* gene were encapsulated in nLRs and grown in CDM with 0.4% D-lactose for 48 h at 30 °C. Diameters of the colonies for each of the strains were measured within the nLRs by bright-field microscopy (n=179 to 194). Strains A11 and G3 form considerably smaller colonies on D-lactose as carbon-source than B3 and C9. The growth parameters of the 4 strains were then extrapolated from the colony-sizes and compared to parameters derived from growth of the strains in liquid culture. Scale bar: 200 µm.

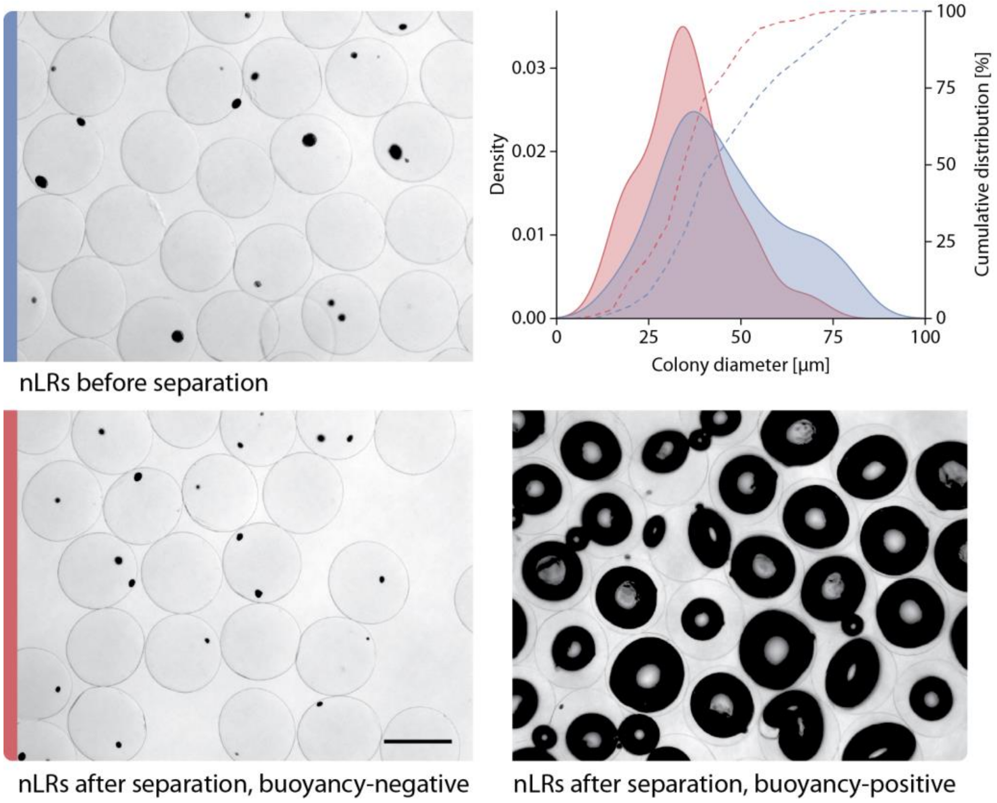

Supplementary Figure 7. Separation of fast- and slow-growing *E. coli*.

A mixture of 18 different variants of *E. coli* strain EcNR1_XX [pCRISPR_lacZ + pCat] carrying a modulated RBS in front of the genomic *lacZ* gene were encapsulated in nLRs and grown with D-lactose as the carbon source for 48 h at 30 °C. Bright-field microscopic analysis was used to determine the colony-size distribution of the total population before (blue labels) and of the bottom (buoyancy-negative) fraction (red labels) after separation. After separation, the nLRs are split into two fractions (lower panel), one at the bottom without gas space in the nLR (left), and one at the top with gas-filled spaces (right picture). The cumulative distribution functions (dashed lines) of the total nLR population before (n=124) and the bottom fraction after separation (n=101) highlight the different colony-size distributions before and after separation and indicating the removal of large colonies from in the buoyancy-negative population. Scale bar: 400 µm.

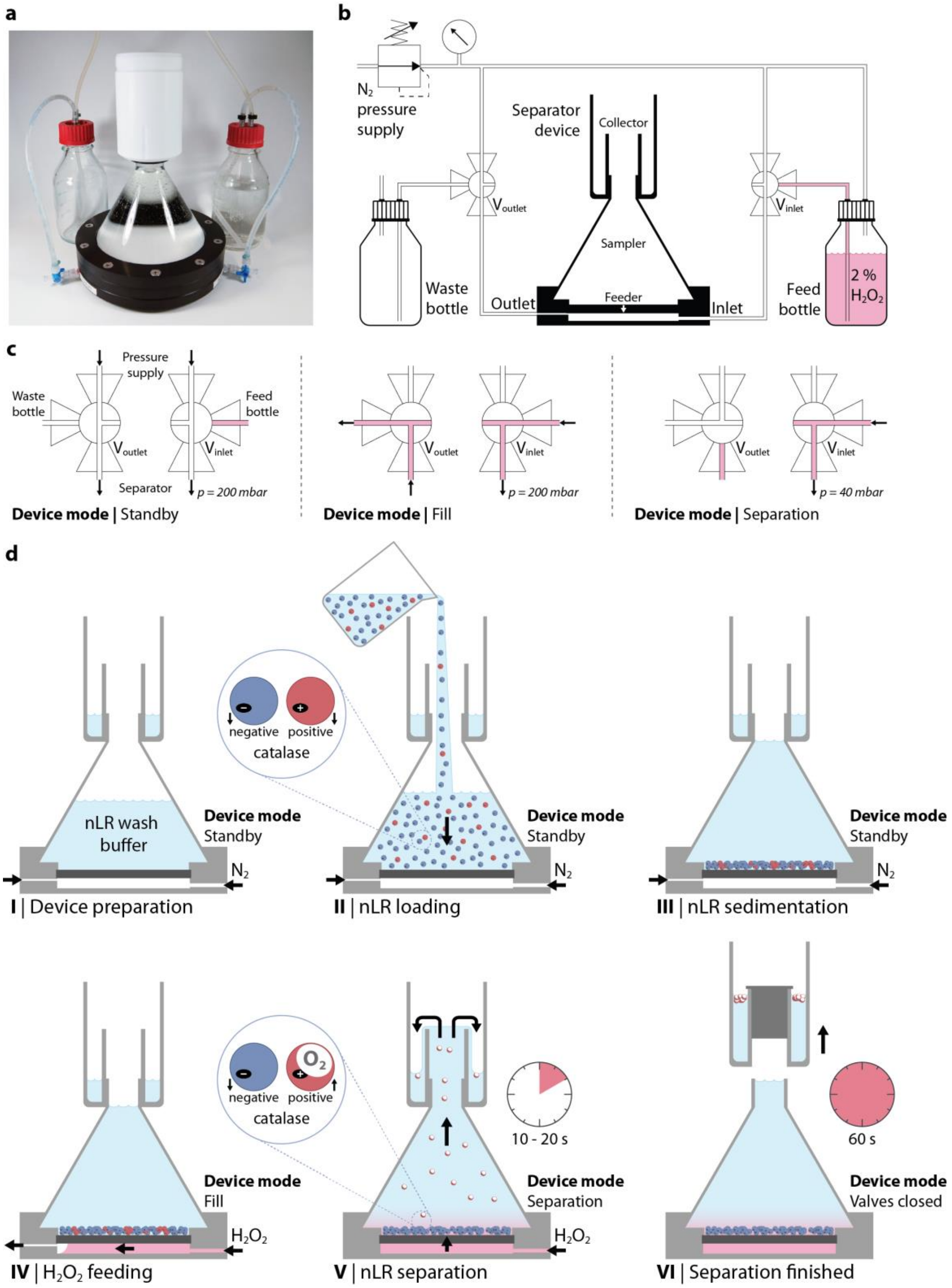

Supplementary Figure 8. Function principle of the separator device.

(a) Photograph of the separator device without tubing and N_2-supply accessories. (b) Connection scheme of the separator

device. The device features three chambers: A sampler, holding the nLRs and facilitating the separation of the nLRs upon H_2O_2 treatment, a collector for the entrapment of floating nLRs during separation, and a feeder for supply of H_2O_2 to the sampler. The sampler is separated from the feeder by a porous bottom and a membrane permeable for liquids but impermeable for gas with pressures below the bubble-point (which is typically higher than 3,000 mbar). The liquid flow within the feeder is regulated via the application of pressure from the nitrogen gas reservoir and two manually operated 3-way valves. Prior to the separation experiment, H_2O_2 (2% in nLR wash buffer) is filled into the feed bottle and connected to the inlet valve (V_{inlet}) of the feeder. A waste bottle, holding the overflow of H_2O_2 during feeding is connected to the outlet valve (V_{outlet}) of the feeder. Note that the positions of the valves in the scheme correspond to the status of the device prior to separation. (c) Different valve positions of the separator device throughout the separation process. H_2O_2 streams are indicated in light red, flow direction to and from the valves are indicated by black arrows. (d) Separation workflow using the separator device. At the beginning of the experiment, the device is in standby mode. Here, the system pressure is regulated to 200 mbar and the feeder is filled with N_2. The overpressure in the feeder prevents leakage of liquid from the sampler into the feeder. The nLR wash buffer is filled into the sampler (approx. 500 mL) and the collector (approx. 50 mL) (I). Next, the nLRs (in 500 mL nLR wash buffer) are poured into the sampler (II). After sedimentation of the nLRs (1 to 5 min, depending on the size of the nLRs) (III), the device is switched to fill mode. Now, the gas in the feeder is replaced by H_2O_2, supplied from the feed bottle (IV). Once the complete chamber is filled with H_2O_2 (indicated by an overflow into the waste bottle) the device is switched into the separation mode and the pressure is reduced to 40 mbar. The H_2O_2 is now fed through the porous bottom into the sampler where it reaches the nLRs. A catalase-positive colony within a nLR lowers its density, causing it to ascend to the upper part of the sampler (V). As the flow of diluted H_2O_2 increases the volume in the device, the sampler unit continuously carries floating nLRs over into the collector. After separation, the inlet and outlet valves are closed, the collector is removed from the device, and the collected nLRs are recovered. Note that the capacity of the separator device is specified by its diameter and the thickness of the nLR after sedimentation (see Supplementary Notes).

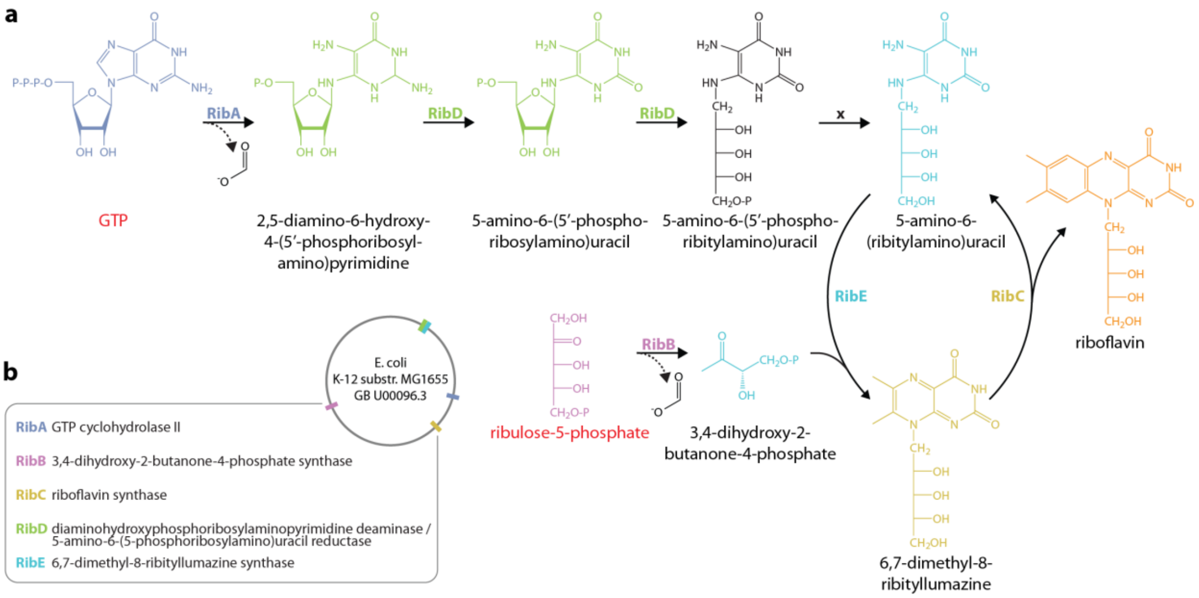

Supplementary Figure 9. B_2 biosynthesis in *E. coli*.

(a) B_2 biosynthesis in *E. coli* is provided from central metabolism with one molecule of GTP and two molecules of D-ribulose-5-phosphate (highlighted in red)[37]. Note that the conversion of 5-amino-6-(5’-phospho-ribitylamino)uracil to 5-amino-6-(ribitylamino)uracil (indicated as “x”) is catalysed by different phosphatases not exclusively dedicated to the B_2 metabolism[38]. (b) Enzymes involved in B_2 biosynthesis and the location of the corresponding genes on the chromosome of *E. coli* MG1655[39].

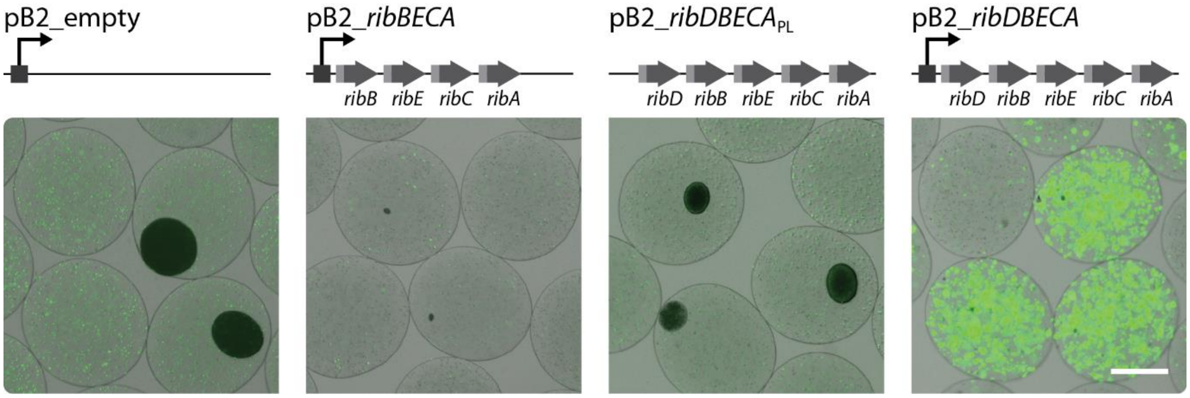

Supplementary Figure 10. B_2 production and sensing in nLRs.

Four variants of *E. coli* BW23474 carrying control or B_2 production plasmids were encapsulated (on average 0.2 cells per nLR) together with sensor cells (on average 1,000 cells per nLR). Microscopic analysis of the nLRs after incubation. Shown are overlays of bright-field and epifluorescence images using the GFP filter-set (see Supplementary Methods). Note that only the strain carrying the full B_2 biosynthesis operon pB2_*ribDBECA* enables expression of the Cat-GFP marker within the nLRs as indicated by green fluorescence of the nLR-embedded sensor cells. Dark spots indicate a non-fluorescent producer colony where a potential metabolic burden resulting from the overexpression of at least one of the pathway enzymes influences the final colony sizes of each variant. Scale bar: 200 µm.

Supplementary References

1. Salsac, A.-V., Zhang, L. & Gherbezza, J.-M. Measurement of mechanical properties of alginate beads using ultrasound. *19eme Congr. Fr. Mec.* 1–6 (2009).

2. Meyenburg, K. V. O. N. & Andersen, K. B. Are growth rates of *Escherichia coli* in batch cultures limited by respiration? *J. Bacteriol.* **144,** 114–123 (1980).

3. Milo, R., Jorgensen, P., Moran, U., Weber, G. & Springer, M. BioNumbers - the database of key numbers in molecular and cell biology. *Nucleic Acids Res.* **38,** 750–753 (2009).

4. Switala, J. & Loewen, P. C. Diversity of properties among catalases. *Arch. Biochem. Biophys.* **401,** 145–154 (2002).

5. Lindskog, S. Structure and mechanism of carbonic anhydrase. *Pharmacol. Ther.* **74,** 1–20 (1997).

6. Brandenberger, H. & Widmer, F. A new multinozzle encapsulation/immobilisation system to produce uniform beads of alginate. *J. Biotechnol.* **63,** 73–80 (1998).

7. Amstad, E. *et al.* Robust scalable high throughput production of monodisperse drops. *Lab Chip* **16,** 4163–4172 (2016).

8. Roberts, T. M. *et al.* Identification and characterisation of a pH-stable GFP. *Sci. Rep.* **6,** 28166 (2016).

9. Powell, K. T. & Weaver, J. C. Gel microdroplets and flow cytometry: rapid determination of antibody secretion by individual cells within a cell population. *Biotechnology* **8,** 333–337 (1990).

10. Walser, M., Leibundgut, R. M., Pellaux, R., Panke, S. & Held, M. Isolation of monoclonal microcarriers colonized by fluorescent *E. coli*. *Cytom. Part A* **73A,** 788–798 (2008).

11. Fischlechner, M. *et al.* Evolution of enzyme catalysts caged in biomimetic gel-shell beads. *Nat. Chem.* **6,** 791–796 (2014).

12. Duarte, J. M., Barbier, I. & Schaerli, Y. Bacterial Microcolonies in gel beads for high-throughput screening of libraries in synthetic biology. *ACS Synth. Biol.* **6,** 1988–1995 (2017).

13. Buffi, N. *et al.* Development of a microfluidics biosensor for agarose-bead immobilized *Escherichia coli* bioreporter cells for arsenite detection in aqueous samples. *Lab Chip* **11,** 2369–2377 (2011).

14. Baba, T. *et al.* Construction of *Escherichia coli* K-12 in-frame, single-gene knockout mutants: the Keio collection. *Mol. Syst. Biol.* **2,** (2006).

15. Thomason, L. C., Costantino, N. & Court, D. L. *E. coli genome manipulation by P1 transduction*. *Current Protocols in Molecular Biology* **Chapter 1,** (John Wiley & Sons, Inc., 2007).

16. Datsenko, K. A. & Wanner, B. L. One-step inactivation of chromosomal genes in *Escherichia coli* K-12 using PCR products. *Proc. Natl. Acad. Sci. U. S. A.* **97,** 6640–6645 (2000).

17. Beers Jr., R. F. & Sizer, I. W. A spectrophotometric method for measuring the breakdown of hydrogen peroxide by catalase. *J. Biol. Chem.* **195,** 133–140 (1952).

18. Mishra, S. & Imlay, J. Why do bacteria use so many enzymes to scavenge hydrogen peroxide? *Arch. Biochem. Biophys.* **525,** 145–160 (2012).

19. Sprouffske, K. & Wagner, A. Growthcurver: an R package for obtaining interpretable metrics from microbial growth curves. *BMC Bioinformatics* **17,** 172 (2016).

20. Shetty, R. P., Endy, D. & Knight, T. F. Engineering BioBrick vectors from BioBrick parts. *J. Biol. Eng.* **2,** 5 (2008).

21. Brinkley, M. A brief survey of methods for preparing protein conjugates with dyes, haptens and crosslinking reagents. *Bioconjug. Chem.* **3,** 2–13 (1992).

22. Blonk, J. C. G., van Eendenburg, J., Koning, M. M. G., Weisenborn, P. C. M. & Winkel, C. A new

CSLM-based method for determination of the phase behaviour of aqueous mixtures of biopolymers. *Carbohydr. Polym.* **28,** 287–295 (1995).

23. Schindelin, J. *et al.* Fiji: an open-source platform for biological-image analysis. *Nat. Methods* **9,** 676–682 (2012).

24. Schindelin, J., Rueden, C. T., Hiner, M. C. & Eliceiri, K. W. The ImageJ ecosystem: an open platform for biomedical image analysis. *Mol. Reprod. Dev.* **82,** 518–529 (2015).

25. Hanahan, D. Techniques for transformation of *E. coli*. *DNA cloning A Pract. Approach* **1,** 109–135 (1985).

26. Posfai, G. Emergent properties of reduced-genome *Escherichia coli*. *Science* **312,** 1044–1046 (2006).

27. Wang, H. H. *et al.* Programming cells by multiplex genome engineering and accelerated evolution. *Nature* **460,** 894–898 (2009).

28. Haldimann, A. *et al.* Altered recognition mutants of the response regulator PhoB: a new genetic strategy for studying protein-protein interactions. *Proc. Natl. Acad. Sci. U. S. A.* **93,** 14361–14366 (1996).

29. Haas, A., Brehm, K., Kreft, J. & Goebel, W. Cloning, characterization, and expression in *Escherichia coli* of a gene encoding *Listeria seeligeri* catalase, a bacterial enzyme highly homologous to mammalian catalases. *J. Bacteriol.* **173,** 5159–5167 (1991).

30. Dykxhoorn, D. M., St. Pierre, R. & Linn, T. A set of compatible *tac* promoter expression vectors. *Gene* **177,** 133–136 (1996).

31. Cherepanov, P. P. & Wackernagel, W. Gene disruption in *Escherichia coli*: TcR and KmR cassettes with the option of Flp-catalyzed excision of the antibiotic-resistance determinant. *Gene* **158,** 9–14 (1995).

32. Jiang, W., Bikard, D., Cox, D., Zhang, F. & Marraffini, L. A. RNA-guided editing of bacterial genomes using CRISPR-Cas systems. *Nat. Biotechnol.* **31,** 233–239 (2013).

33. Link, A. J., Phillips, D. & Church, G. M. Methods for generating precise deletions and insertions in the genome of wild-type *Escherichia coli*: application to open reading frame characterization. *J. Bacteriol.* **179,** 6228–6237 (1997).

34. Meyer, A. *et al.* Optimization of a whole-cell biocatalyst by employing genetically encoded product sensors inside nanolitre reactors. *Nat. Chem.* **7,** 673–678 (2015).

35. Silva-Rocha, R. *et al.* The Standard European Vector Architecture (SEVA): A coherent platform for the analysis and deployment of complex prokaryotic phenotypes. *Nucleic Acids Res.* **41,** D666–D675 (2013).

36. Bosshart, A., Hee, C. S., Bechtold, M., Schirmer, T. & Panke, S. Directed divergent evolution of a thermostable D-tagatose epimerase towards improved activity for two hexose substrates. *ChemBioChem* **16,** 592–601 (2015).

37. Bacher, A., Eberhardt, S., Fischer, M., Kis, K. & Richter, G. Biosynthesis of vitamin B2 (riboflavin). *Annu. Rev. Nutr.* **20,** 153–167 (2000).

38. Haase, I. *et al.* Enzymes from the haloacid dehalogenase (HAD) superfamily catalyse the elusive dephosphorylation step of riboflavin biosynthesis. *ChemBioChem* **14,** 2272–2275 (2013).

39. Blattner, F. R. The complete genome sequence of *Escherichia coli* K-12. *Science* **277,** 1453–1462 (1997).
